# CRISPR-Cas9 editing of TLR4 to improve the outcome of cardiac cell therapy

**DOI:** 10.1038/s41598-023-31286-4

**Published:** 2023-03-18

**Authors:** Yeshai Schary, Itai Rotem, Tal Caller, Nir Lewis, Olga Shaihov-Teper, Rafael Y. Brzezinski, Daria Lendengolts, Ehud Raanani, Leonid Sternik, Nili Naftali-Shani, Jonathan Leor

**Affiliations:** 1 Neufeld and Tamman Cardiovascular Research Institutes, Sheba Medical Center, Sackler School of Medicine, Tel Aviv University, Tel Aviv, Israel; 2 Heart Center, Sheba Medical Center, 52621 Tel-Hashomer, Israel; 3 Department of Cardiac Surgery, Leviev Cardiothoracic and Vascular Center, Sheba Medical Center, Sackler School of Medicine, Tel Aviv University, Tel Aviv, Israel

**Keywords:** Cardiovascular biology, Mesenchymal stem cells, Cardiovascular models

## Abstract

Inflammation and fibrosis limit the reparative properties of human mesenchymal stromal cells (hMSCs). We hypothesized that disrupting the toll-like receptor 4 (TLR4) gene would switch hMSCs toward a reparative phenotype and improve the outcome of cell therapy for infarct repair. We developed and optimized an improved electroporation protocol for CRISPR-Cas9 gene editing. This protocol achieved a 68% success rate when applied to isolated hMSCs from the heart and epicardial fat of patients with ischemic heart disease. While cell editing lowered TLR4 expression in hMSCs, it did not affect classical markers of hMSCs, proliferation, and migration rate. Protein mass spectrometry analysis revealed that edited cells secreted fewer proteins involved in inflammation. Analysis of biological processes revealed that TLR4 editing reduced processes linked to inflammation and extracellular organization. Furthermore, edited cells expressed less NF-ƙB and secreted lower amounts of extracellular vesicles and pro-inflammatory and pro-fibrotic cytokines than unedited hMSCs. Cell therapy with both edited and unedited hMSCs improved survival, left ventricular remodeling, and cardiac function after myocardial infarction (MI) in mice. Postmortem histologic analysis revealed clusters of edited cells that survived in the scar tissue 28 days after MI. Morphometric analysis showed that implantation of edited cells increased the area of myocardial islands in the scar tissue, reduced the occurrence of transmural scar, increased scar thickness, and decreased expansion index. We show, for the first time, that CRISPR-Cas9-based disruption of the TLR4-gene reduces pro-inflammatory polarization of hMSCs and improves infarct healing and remodeling in mice. Our results provide a new approach to improving the outcomes of cell therapy for cardiovascular diseases.

## Introduction

The inconclusive results of cell therapy trials for heart disease^1–3^ drive the search for alternative strategies. Mesenchymal stromal cells (MSCs), also known as mesenchymal stem cells, possess immunomodulatory, anti-inflammatory, and reparative properties^4–6^, either directly or via the release of free cytokines and extracellular vesicles (EVs)^7^. MSCs have emerged as a viable source for cardiac cell therapy^6,8^. However, the environment of the diseased heart may drive resident and transplanted MSCs toward a pro-inflammatory phenotype and restrict their survival and reparative effects^9–11^. In part, this effect is mediated by toll-like receptor 4 (TLR4)^9,10^, a membranous and endosomal receptor that controls the innate immune system by recognizing a broad spectrum of molecules, such as damage-associated molecular patterns (DAMPs)^12,13^. The phenomenon of pro-inflammatory MSCs aligns with the new paradigm of MSC polarization^14^. The primary mechanism of pro-inflammatory polarization is mediated by TLR4^14^.

The introduction of clustered regularly interspaced short palindromic repeats (CRISPR) as a gene-editing tool has revolutionized basic and translational research^15–17^. CRISPR can be applied to either disrupt, knockout (KO) or knock-in genes^18^. However, the clinical application of CRISPR technology in vivo has been restricted by efficacy and safety concerns^15,18^. Here, we aimed to test the hypothesis that ex vivo disruption of the TLR4 gene by the CRISPR-Cas9 system will improve the reparative properties of cardiac MSCs. The strategy of ex vivo gene editing of TLR4 is clinically relevant and can improve the outcome of cell therapy for heart repair.

## Results

### Electroporation-based CRISPR-Cas9 TLR4 gene editing

**Figure 1:**
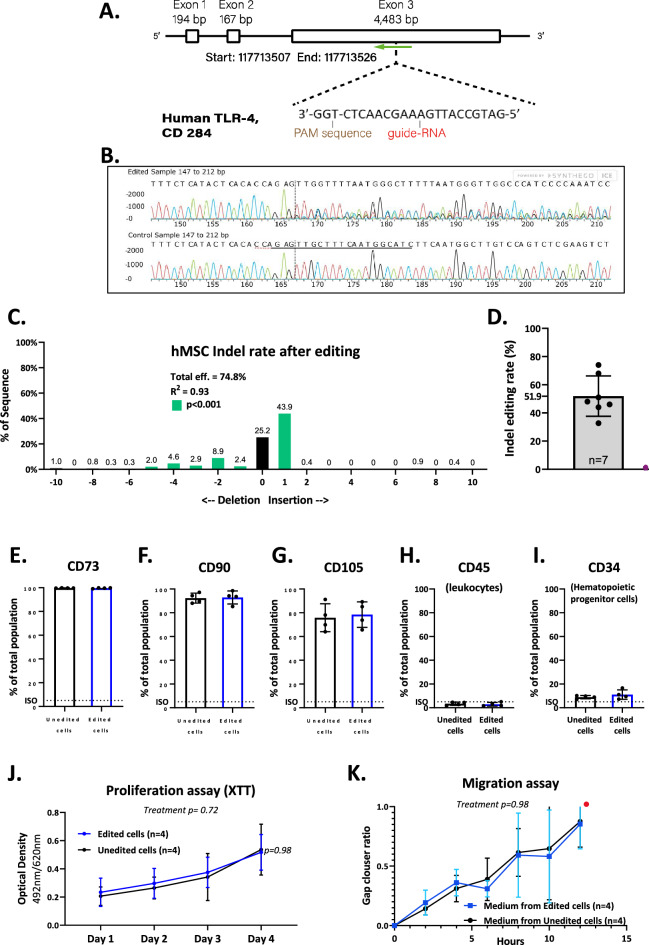
CRISPR-Cas9 TLR4 Editing in Human MSCs. (**A**) To edit the TLR4 gene in hMSCs we designed a sgRNA targeting exon III. (**B**) To calculate the editing efficiency, we produced genomic DNA from the cells and sequenced the target site with Sanger sequencing, with and without gene editing, marked by the horizontal dotted line. (**C**) For each experiment, we calculated the indel rate with indel distribution analysis (TIDE). (**D**) Average editing efficiency (%) with cells from seven different patients. We used 4 × 10^5^ cells and read 1 × 10^5^ for each sample. Positive (**E**–**G**) and negative (**H**,**I**) hMSC surface markers show no differences in the expression levels between the edited and unedited cells after comparison to a matching isotype control, as found by flow cytometry. Statistical analyses by unpaired two-tailed t-test, n = 4. (**J**) To determine the effect of gene editing on hMSC proliferation and viability, we used a proliferation assay (XTT). hMSCs (5 × 10^4^ per well, in duplicates) were incubated for four consecutive days. Gene editing did not affect cell proliferation. Statistical analysis by the mixed-effects model (REML) with Holm-Šídák's post-test, n = 4. (**K**) We used a migration scratch assay to determine whether disrupting the TLR4 gene affects hMSC migration. We scratched sheets of cultured hMSCs. Next, we exposed the hMSCs to conditioned media from edited and unedited cells. Gene editing did not affect the cell migration rate. Statistical analysis by two-way ANOVA with Holm-Šídák's post-test, n = 4. *Abbreviations* hMSCs, human mesenchymal stromal cells; indel, insertion and deletion; PBS, phosphate-buffered saline; sgRNA, single guide RNA; XTT, 2,3-bis-(2-methoxy-4-nitro-5-sulfophenyl)-2H-tetrazolium-5-carboxanilide.

To disrupt the expression of the TLR4 gene in human cardiac MSCs (hMSCs), we developed an improved electroporation protocol. Donor patients’ basic clinical characteristics are available in Supplementary Table 3. We used a TLR4-targeted ribonucleoprotein Cas9. We started by comparing three single guide RNAs (sgRNAs) located in exon I. The general yield of these sgRNAs was around 30%. To further increase the insertion and deletion (indel) rate and to prevent alternative splicing of the gene, we added another sgRNA to the comparisons that target exon III on the antisense strand of the DNA. This sgRNA was highly efficient and thus used for the rest of the experiments (Fig. 1A)^19^. Unedited control groups were subjected to the same editing protocol but with scrambled sgRNA. Two days after the procedure, we sequenced the target site and aligned the DNA sequence, comparing edited and unedited cells at the cut site of the DNA (Fig. 1B). Using the tracking of indels by decomposition (TIDE) algorithm^20^, we calculated the distribution and rate of nucleotide indels in the sequence (Fig. 1C). Further sequencing data analysis showed that adding one nucleotide was the most abundant repair event (43.9%, mostly cytosine, Fig. 1C). Seven replicate reactions showed a 51.9% indel rate that translated into frameshifts in the reading frame (Fig. 1D). A minimal indel rate of > 50% was set for all further experiments. Overall, our electroporation protocol achieved a high, robust percentage of editing in primary human cells in a simple procedure.

### The effect of TLR4 gene editing on hMSC phenotype

To determine the effect of TLR4 gene editing on hMSC phenotype, we assessed the expression of hMSC markers by flow cytometry. Markers for hMSCs were maintained after TLR4 gene editing: CD73^+^ (99%), CD90^+^ (93%), and CD105^+^ (77%), (Fig. 1E–G). In addition, both edited and unedited hMSCs exhibited low expression of hematopoietic lineage markers: CD45^−^ (< 5%) and CD34^−^ (10%) (Fig. 1H,I).

Next, we sought to determine the effect of TLR4 editing on the functional characteristics of the edited hMSCs. Gene editing did not affect the hMSC proliferation rate or viability over 4 days, as indicated by the metabolic-based XTT assay (Fig. 1J), nor hMSC migration rate, as indicated by the "scratch" assay (Fig. 1K, Supplementary Figure 1A). Overall, electroporation-based editing of the TLR4 gene using Cas9 RNPs did not affect the expression of hMSC markers and the rate of proliferation and migration.

### TLR4 expression in hMSCs after editing

**Figure 2:**
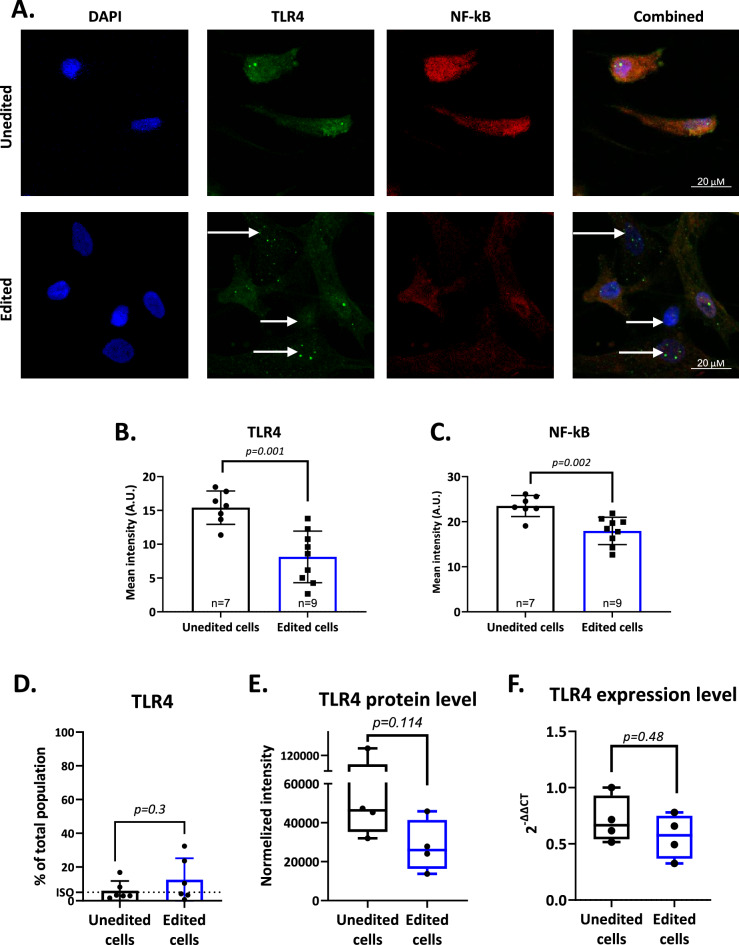
Effect of TLR4 Gene Disruption on Human MSCs. (**A**) Representative immunofluorescent images of hMSCs with anti-TLR4 and anti-NF-ƙB antibodies. White arrows indicate edited cells with little expression of TLR4 and no visible NF-ƙB after editing. (**B**,**C**) Quantification of TLR4 and NF-ƙB staining in hMSCs following TLR4 gene editing using ImageJ 1.53c, http://imagej.nih.gov/ij^72^. Each sample represents one field of view acquired from three replicates in two separate experiments. Analysis of images shows a reduction of TLR4 expression after editing and less NF-ƙB expression. P-values by Mann–Whitney test. (**D**) TLR4 expression was measured by FACS and showed no significant signal over isotype read in both groups. Considering that no membrane permeabilization was done, we posit this was likely due to the internalization of the TLR4. (**E**) Using western blot quantification, we found a 50% decrease in TLR4 protein levels. Cropped images as well as full raw images of data can be found in Supplementary Figure 2A. (**F**) Gene expression analysis by qRT-PCR revealed no significant differences in the levels of mRNA with and without editing. Statistical analyses for all TLR4 comparisons by Mann–Whitney test, n = 4. *Abbreviations* hMSCs, human mesenchymal stromal cells; TLR4, toll-like receptor 4; NF-ƙB, nuclear factor-kappa B; FACS, flow cytometry; mRNA, messenger ribonucleic acid; qRT-PCR, quantitative reverse transcription-polymerase chain reaction.

We used several assays to determine the effect of TLR4 gene editing on TLR4 protein expression. Immunostaining of TLR4 and its primary target, the transcription factor NF-ƙB, in edited compared to unedited hMSCs revealed a 52% decrease in the mean intensity of expression of TLR4 (Fig. 2B, 15.4 vs. 8.1, *p* = 0.0012) and a 76% decrease in the mean intensity of NF-ƙB (Fig. 2C, 23.4 vs. 19.1, *p* = 0.0021) (Fig. 2A–C, Supplementary Figure 1B). In contrast to the high abundance of TLR4, we found that by immunostaining the surface, TLR4 was undetectable by flow cytometry in both edited and unedited cells (Fig. 2D). Therefore, we assumed that TLR4 was translocated intracellularly in inflamed hMSCs. The latter finding confirms that other cell types can internalize TLR4 into the cytoplasm^21,22^. Overall, TLR4 targeting by CRISPR-Cas9 reduced one of the main downstream signaling mechanisms of inflammatory pathways.

Gene editing reduced the protein level of TLR4 by 56%, measured by western blot (Fig. 2E, Supplementary Figure 2A). In contrast, gene editing did not significantly affect the expression of TLR4 mRNA (Fig. 2F). These conflicting findings may reflect the consequence of gene disruption at exon III. The TLR4 gene was translated into mRNA, but the mRNA was insufficient for synthesizing a functional TLR4 protein. Thus, disruption of the TLR4 gene by CRISPR-Cas9 reduced the expression of TLR4.

### TLR4 gene editing modulated hMSC secretome

**Figure 3:**
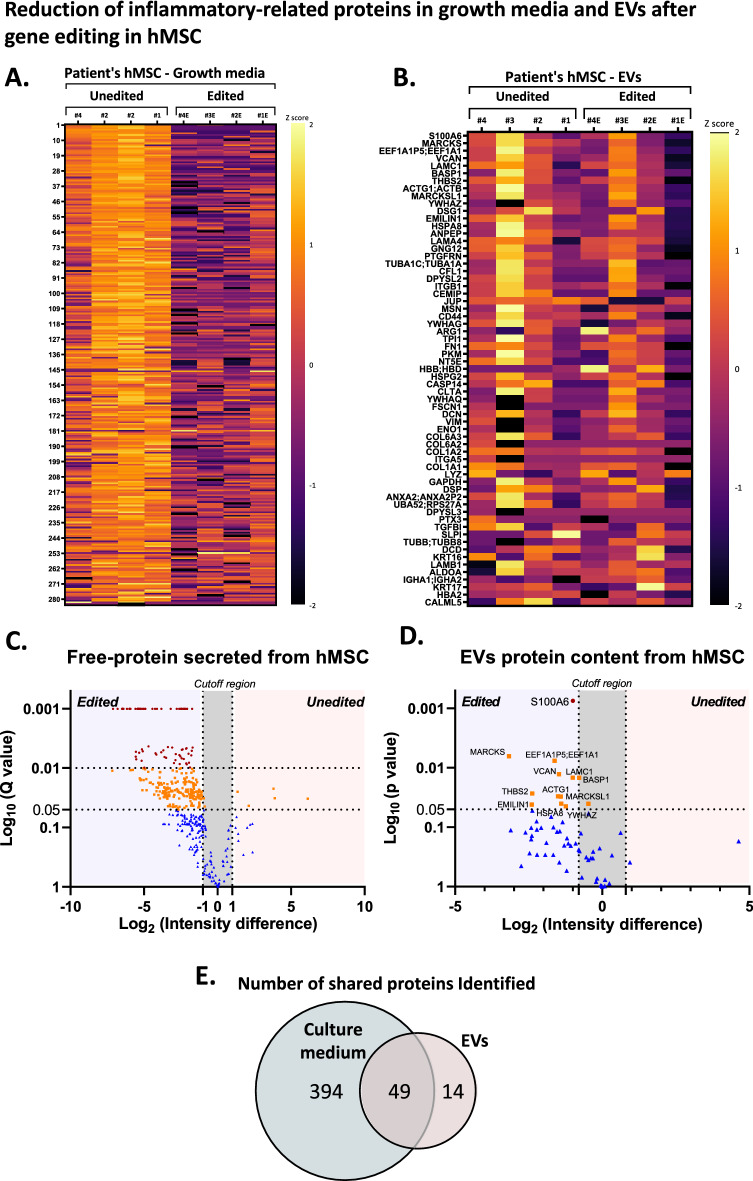
Proteomics Analysis Indicated that Disrupting the TLR4 Gene Reduced Secretion of Pro-Inflammatory Proteins. To determine differences in the proteome of free proteins in growth media and purified EVs released from edited and unedited hMSCs, we carried out a comparative MS proteomic analysis. Data are available via ProteomeXchange with identifier PXD033253. (**A**) Heat map showing the levels of proteins secreted by hMSCs with and without TLR4 gene editing from four patients shown as Z-scores (abundance between -2 and 2). (**B**) Heat map of EV-encapsulated proteins in EVs secreted by hMSCs with and without gene editing of cells from four patients shown as Z-scores (abundance between -2 and 2). (**C**) To graphically present the quantitative data, we constructed a volcano plot (log_2_ fold-change edited vs. unedited cells, plotted against log_10_ of statistical difference). For free-protein secretion, q-value was used to determine the statistical strength of protein identification, with ±1 as the cutoff region for significant changes in secretion after editing. (**D**) For EV protein content, the *p* value was used for statistical strength of protein identification, with ±0.8 as the cutoff region of significant changes. Points above the non-axial vertical line at 0.05 represent proteins with significantly different abundances (*p* < 0.05). Results show a significant reduction in the release of proteins involved in immune regulation (red) and extracellular organization pathways (orange). n = 4. (**E**) The number of validated protein signatures found in each experimental group, separately and combined. The complete list of protein names is specified in Supplementary Table 1. *Abbreviations* TLR4, toll-like receptor 4; hMSCs, human mesenchymal stromal cells; EVs, extracellular vesicles; FBS, fetal bovine serum; MS proteomics, mass spectrometry proteomics.

Paracrine properties are a major determinant of the reparative and immunomodulatory effects of MSCs^6,23,24^. Thus, we conducted a proteomic analysis to determine the effect of TLR4 gene editing on the hMSC secretome. Proteomic analysis was conducted on conditioned media and EVs from edited and unedited hMSCs from four patients. Nano-tracking analysis showed that the size distribution of EVs was smaller than 200 nm, with smaller EVs derived from edited cells (Supplementary Figure 2B). We found that TLR4 editing decreased the secretion of both soluble (Fig. 3A,C) and EV-encapsulated proteins (Fig. 3B,D). To analyze quantitative data of proteins identified by proteomics, we created a Z-score grade for each protein based on expression level. Comparing proteins secreted into the conditioned media from all four patients’ cell lines showed that all had a similar protein profile before and after editing. Moreover, a significant decrease in the amount of secreted proteins was found after editing (Fig. 3A). Unlike the free soluble proteins, analysis of EV proteomics showed high diversity between patients’ cell lines. Even so, the average change after editing the cells was a significant decrease in the level of secreted proteins (Fig. 3B).

To provide further insights, we rearranged the proteomic data on a logarithmic scale according to significance of change (Fig. 3C,D). We found that editing hMSCs reduced the secretion of regulatory proteins from the immune system and the extracellular matrix (ECM) (Supplementary Table 1).

Analysis of EV-encapsulated proteins identified a total of 63 proteins. Of them, 14 were unique to EVs, compared to the conditioned medium (Fig. 3E, Supplementary Table 1). Furthermore, disrupting the TLR4 gene significantly lowered the expression of 13 EV-encapsulated proteins. Amongst them, the protein with the lowest expression after gene editing was the heat-shock protein S100A6 (Fig. 3D), an activator of TLR4 (q < 0.0098)^25^. Overall, our data showed that disruption of the TLR4 gene affects the hMSC secretome.

**Figure 4:**
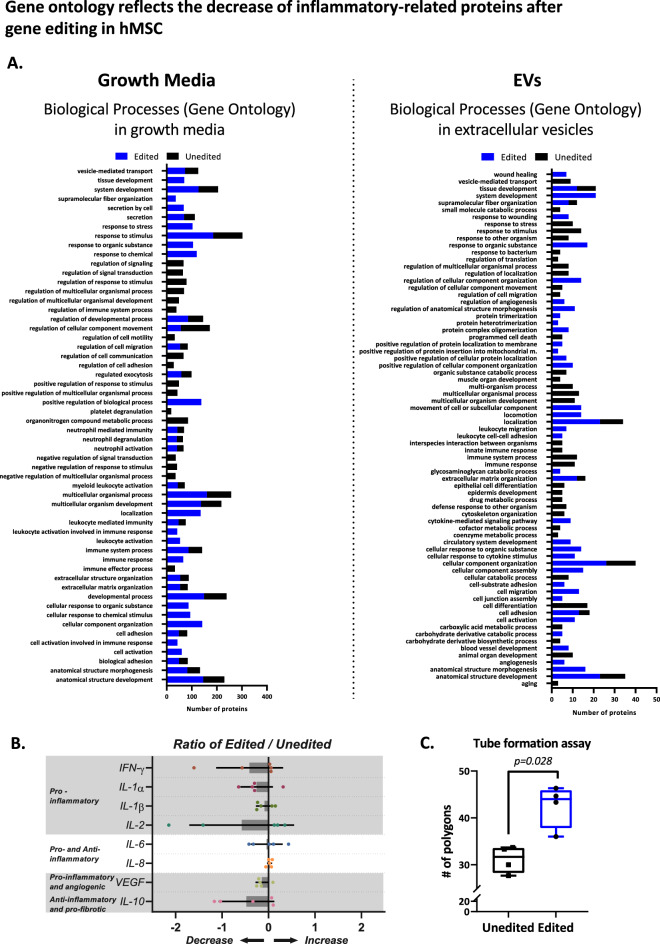
Change in Biological Pathways after Gene Editing in hMSCs. To interpret the changes in the proteomic data before and after gene editing, we clustered the proteins from growth media or EVs by the STRING classification system (https://string-db.org). For each protein group, datasets of protein lists were composed based on the significance of the change. Proteins that had a significant decrease (*p* < 0.05) were grouped into the list of edited cells group, while proteins with no significant change comprised the second list, representing the unedited cells group. (**A**) STRING classification of the 20 most significant biological processes from edited and unedited protein data sets. False-discovery rate according to *p* values corrected for multiple testing by Benjamini–Hochberg. (**B**) Multiplex ELISA analysis of inflammatory cytokine levels in hMSC growth media of edited vs. unedited cells. Results show lower secretion of pro-inflammatory cytokines from edited hMSCs. Media was collected from 2 × 10^4^ cells after incubation for three consecutive days. The ratio was calculated for each patient's edited/unedited cells to find changes in cytokine secretion (n = 5). (**C**) To determine the angiogenic properties of the edited cell conditioned medium, we used HDMEC Matrigel Tube Formation Assay. The results were quantified after 3.5 h. *Abbreviations* EVs**,** extracellular vesicles; hMSCs**,** human mesenchymal stromal cells; ELISA**,** enzyme-linked immunosorbent assay; HDMEC**,** human dermal microvascular endothelial cells.

Using advanced analysis of protein–protein interaction networks by STRING^26,27^, we enlisted the 20 most important biological processes that stem from the data of both the growth media and the EVs (Fig. 4A). Analysis of biological processes revealed that TLR4 editing attenuated processes related to inflammation and ECM organization. The immunomodulatory effect of TLR4 editing was apparent in both free and EV-encapsulated proteins.

Next, we analyzed inflammatory-related cytokines directly secreted from the cells using ELISA. We found that, consistent with the proteomic results, the release of pro-inflammatory cytokines such as IL-1α, IL-1β, and IFN-γ was decreased, along with VEGF and the pro-fibrotic, anti-inflammatory cytokine IL-10. Gene editing did not affect the secretion of other cytokines with dual effects on inflammation, such as IL-6 and IL-8 (Fig. 4B).

Finally, conditioned medium from edited hMSCs stimulated a pro-angiogenic response as indicated by endothelial cell tube formation assay (by 1.4-fold compared to unedited cells, *p* = 0.028) (Fig. 4C, Supplementary Figure 2C). Thus, TLR4 editing improved the pro-angiogenic properties of hMSCs in a VEGF-independent manner^28^. Together, our analysis of edited hMSCs secretome suggests that TLR4 editing in hMSCs activated anti-inflammatory and reparative properties.

### Edited hMSCs improved survival, cardiac remodeling, and function after MI

**Figure 5:**
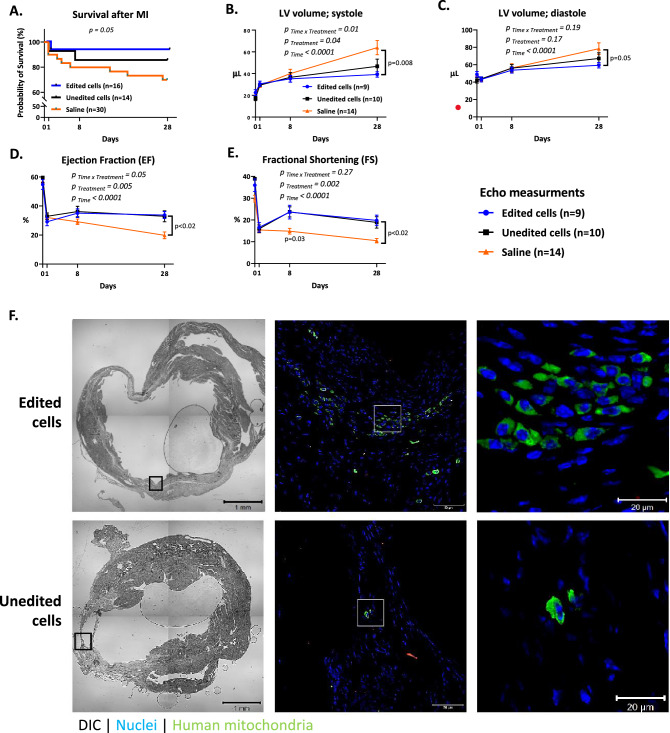
Cell Therapy after MI Improved Survival and Prevented LV Dilatation. To determine the reparative effects of edited and unedited cells on the infarcted heart, we allocated 48 12-week-old Balb/C female mice to MI and a single intra-myocardial injection of edited, unedited cells, or saline. LV remodeling and function were assessed by echocardiography before (baseline), 1, 8, and 28 days after MI. On day 28 post-MI, hearts were harvested for further analyses. (**A**) Survival curve for three treatment groups, 28 days post-MI. Log-rank test for trend analysis demonstrates that mice treated with edited cells had the best survival rate compared with other groups with MI. (**B**,**C**) Serial measurements of systolic and diastolic LV volumes revealed that both edited and unedited cells reduced LV dilatation 28 days after MI, compared with the saline-treated group. This favorable effect was statistically significant for the edited cells. (**D**,**E**) LV contractility, as indicated by EF and FS, was improved by both edited and unedited cells. This favorable effect was statistically significant for the edited cells. Statistical analyses were performed by repeated-measures two-way ANOVA with Holm-Šídák's multiple comparisons test. (**F**) To visualize cell engraftment after injection into the infarct, slides were stained with a human mitochondria marker 28 days post-MI. Images acquired with a confocal microscope show batches of human cells adjacent to the viable myocardial islands within the scar in mice treated with edited cells. On the contrary, only sporadic cells were found in mice treated with unedited cells. *Abbreviations* MI, myocardial infarction; LV, left ventricular; DIC, differential interference contrast; echo, echocardiography; EF, ejection fraction; FS, fractional shortening; hMSCs, human mesenchymal stromal cells.

To test the effect of the edited hMSCs on infarct healing and repair, we subjected mice to MI and cell therapy immediately after infarction. During 28 days of follow-up, edited and unedited hMSCs improved survival (90% and 80%, compared with 70% in saline controls; *p* = 0.05 for edited cells) (Fig. 5A).

Serial echocardiography studies showed that edited and unedited cells improved cardiac remodeling, compared with saline control, 28 days after MI, as indicated by left ventricular (LV) systolic and diastolic volumes (Fig. 5B,C). These favorable effects were statistically significant for the edited cells. Both edited and unedited cells improved LV function, as indicated by LV ejection fraction (EF) and fractional shortening (FS), compared with saline control (Fig. 5D,E). Echocardiography variables before and after MI are available in Supplementary Table 2.

Overall, our echocardiography results indicated that edited hMSCs improved cardiac remodeling and function in mice after MI. Despite a small advantage for edited cells, the favorable effects of edited and unedited hMSC were similar.

### Edited hMSCs improved infarct repair and scar formation

To evaluate the outcome of implanted hMSCs, we stained the mouse heart sections for human mitochondria 28 days after MI (Fig. 5F). In hearts treated with edited cells, we identified clusters of hMSCs. The human cells were located in the scar tissue, near islands of viable myocardium (Fig. 5F, upper panel, in green). Contrary to mice treated with edited cells, only infrequent human cells were found in the hearts of mice treated with unedited hMSCs (Fig. 5F, lower panel). Thus, TLR4 editing improved cell engraftment after MI.

**Figure 6:**
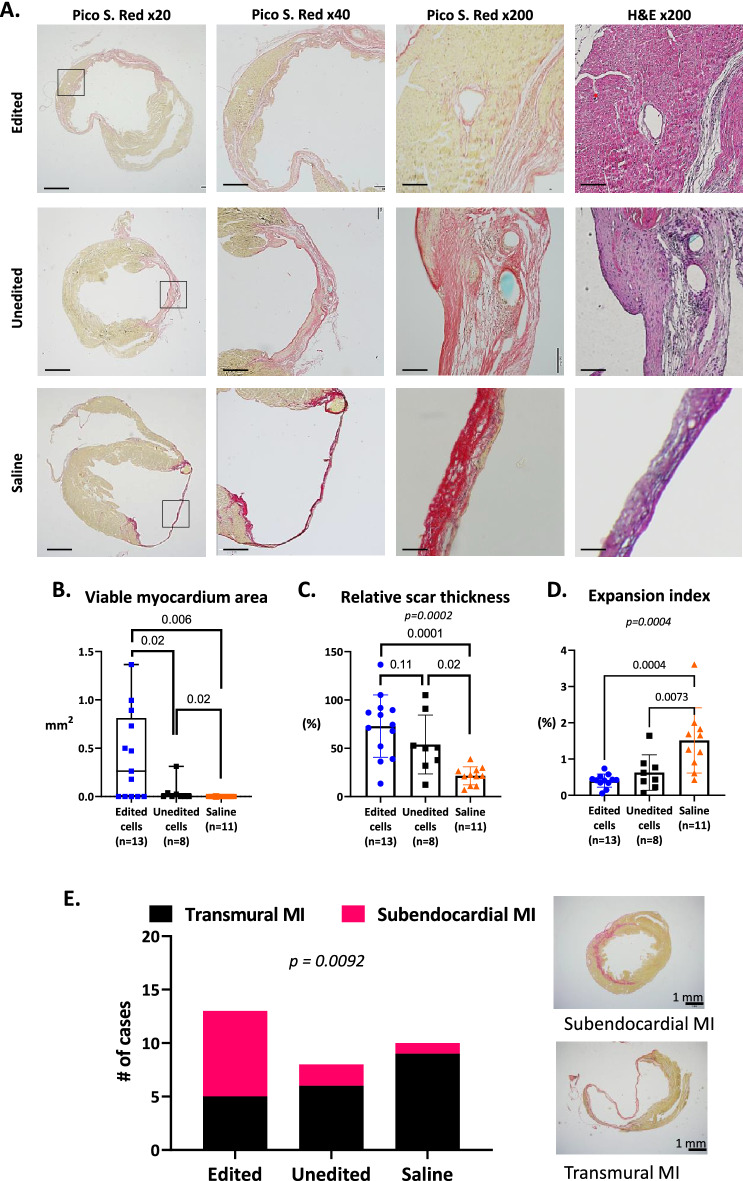
Edited-Cell Therapy in Mice after MI Increased Viability, Preserved Scar Thickness, and Lowered Expansion Index. (**A**) We performed postmortem morphometric analysis 28 days after MI. The slides were stained with hematoxylin–eosin or picrosirius red, photographed, and analyzed with planimetry software (SigmaScan Pro 5, from Systat Software, Inc., San Jose California USA, www.systatsoftware.com). Bar graphs represent: × 20—1 mm; × 40—500 µm; × 200—100 µm; (**B**–**D**) We measured the viable myocardium directly by manually circling the surface area within the scar tissue area. We measured LV maximal diameter, defined as the longest diameter perpendicular to a line connecting the insertions of the septum to the ventricular wall, average wall thickness from three measurements of septum thickness, average scar thickness from three measurements of scar thickness, LV cavity area, and whole LV area. Relative scar thickness and expansion index were calculated as: ${\text{Relative}}\,{\text{scar}}\,{\text{thickness}} = \frac{Average\,scar\,thickness}{{Average\,wall\,thickness}} \times 100.$ ${\text{Expansion}}\,{\text{index}} = \frac{{\frac{LV\,cavity\,area}{{Whole\,LV\,area}}}}{Relative\,scar\,thickness} \times 100.$ Measurements show that only edited hMSCs protected viable myocardium within the scar tissue. They also show thicker scar tissue and smaller expansion index in the hearts of mice that received edited cells. P-values were calculated by one-way ANOVA with Holm-Šídák's multiple comparisons test. (**E**) Further morphological analysis revealed that edited-cell therapy increased the occurrence of a subendocardial, rather than a transmural, scar. *p* value by Chi-squared test for trend. *Abbreviations* MI, myocardial infarction; LAD, left anterior descending; LV, left ventricular; hMSCs, human mesenchymal stromal cells.

Next, to determine the effect of TLR4 editing on infarct repair, we evaluated heart sections 28 days after MI. A unique finding was that islands of viable myocardium were present within the scar tissues of hearts treated with edited cells but not in hearts treated with unedited cells or saline (Fig. 6A). The islands of viable myocardium (upper panel) within the scar tissue are a significant finding, especially when compared to hearts treated with edited hMSCs using manual surface measurements (Fig. 6B). Further histological analysis revealed cell infiltrates at the sites of the scar tissue and particularly in hearts treated with unedited cells, which indicates active inflammation and, most likely, an immune reaction to the human cells (Fig. 6A, middle panel). Significantly, edited, and unedited cells increased scar thickness, as calculated by the average scar thickness divided by the average wall thickness (Fig. 6C). This is a significant outcome because scar thickening reduces wall stress (as defined by the law of Laplace), infarct dyskinesis, and LV dilatation. In this line of evidence, edited hMSCs also markedly reduced the expansion index ([LV cavity area/whole LV area]/relative scar thickness), an indication of adverse LV remodeling, compared with the unedited and control groups (Fig. 6D). Furthermore, treatment with edited hMSCs reduced the incidence of the more severe form of MI, the transmural scar, compared with unedited and saline treatments (38% vs. 75% and 90%; *p* = 0.009) (Fig. 6E). Overall, our histologic and morphometric analyses indicated that treatment with TLR4-edited hMSCs improved infarct repair and scar formation.

## Discussion

**Figure 7:**
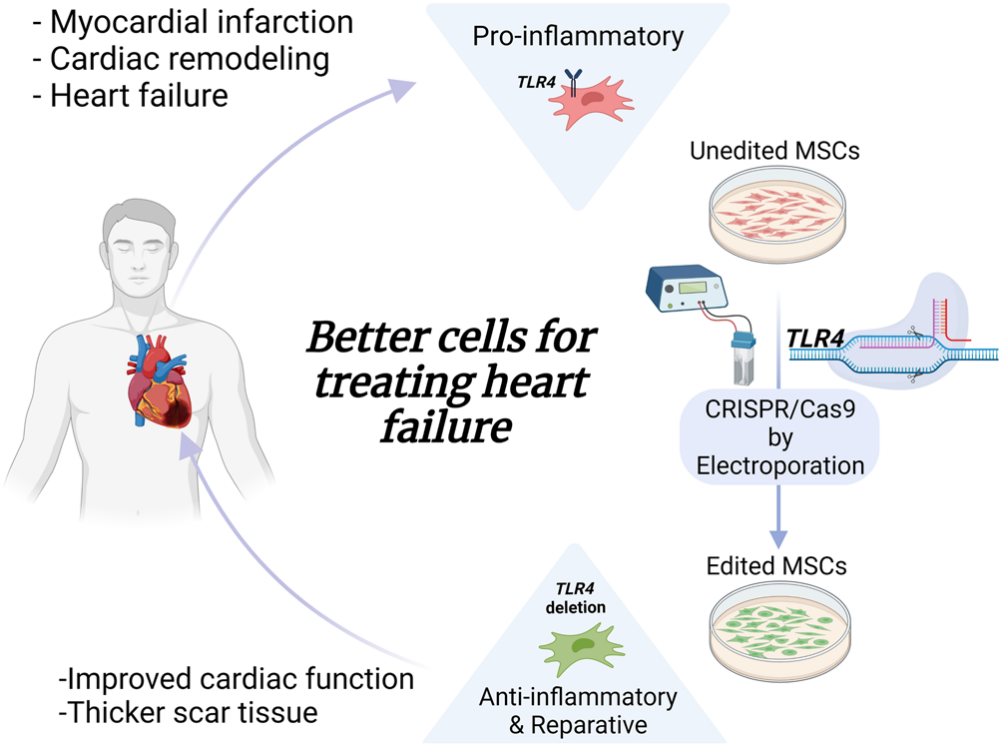
Graphical abstract: genome editing to improve the outcome of cardiac cell therapy. Schematic description of the use of genome editing to improve the outcome of cardiac cell therapy. TLR4 disruption can improve the reparative properties of autologous hMSCs in patients with heart disease. Created with BioRender.com.

Our work provides several new findings. First, we show, for the first time, that CRISPR-based TLR4 disruption in hMSCs improves the function of cells and the outcome of cell therapy after MI. Second, we optimized an improved electroporation protocol to refine CRISPR-based gene editing in human cells. Third, we confirmed and extended upon our previous report^10^, and showed that TLR4 disruption switched hMSCs from patients with ischemic heart disease to an anti-inflammatory and reparative phenotype. Implantation of the edited hMSCs into the infarcted myocardium of mice ameliorated infarct repair and scar formation. Together, we proved that CRISPR-based gene editing could be used to engineer hMSCs with improved therapeutic properties (Fig. 7).

### Targeting TLR4 to enhance the therapeutic properties of hMSCs

Following MI, dying cells and ECM fragments release DAMPs that activate a family of pattern recognition receptors (TLRs), which in turn activate the innate immune system^19,25^. In the infarcted myocardium, the cell surface TLR4 has been implicated in the extension of ischemic injury and the development of adverse remodeling^29–33^. MSCs express functional TLR4^34,35^, and here we have shown that activated TLR4 is localized intracellularly. MSCs can be polarized into a pro-inflammatory and anti-inflammatory phenotype by ligands of TLRs, leading to respective changes in their immunomodulatory and reparative properties^36–39^.

Though previously assumed that TLR4 in hMSCs acts as a transmembrane receptor^19^, we found that in hMSCs TLR4 is located intracellularly, at least part of the time. This is consistent with previous reports^40–42^ of different cell types as well. It is also known that after internalization of TLR4, an LPS signal can still affect inflammatory pathways^43,44^. We believe that the location of TLR4 does not affect its function, and that the receptor can still be activated and promote inflammatory pathways. With regards to the level of the downstream signals of TLR4, there is a baseline level of activation in the cells. Considering the source of the cells in human patients with ischemic diseases, we have previously shown that the cells are already activated and switched toward a pro-inflammatory state^45^. Considering this fact, it is not surprising that by deletion of TLR4, the level of NF-kB stimulation decreases. In the present work, we show that TLR4 deficiency in hMSCs improves cell survival and LV function after MI in mice.

A unique finding in our study was the presence of islands of viable myocardium in the scar tissue of hearts treated with edited hMSCs after MI. This finding was associated with increased scar thickness and reduced expansion index. Based on previous reports^46–48^, it is likely that the edited hMSCs secreted protective factors that improve cardiomyocyte survival and viability at an early stage after infarction.

### Efficient electroporation-based gene editing in hMSCs

CRISPR technology offers new opportunities in the arena of gene editing^15^. The development of an ex vivo platform that combines precise genome editing in vitro with practical application in vivo can improve the outcome of cardiac cell therapy. Trade secrets cover many of the electroporation protocols for CRISPR-based gene editing. As a result, researchers cannot reproduce, adjust, tune, and perfect existing protocols. This lack of transparency may add to the reproducibility challenges in science, and hinders other researchers from establishing new and innovative research. We report our new and efficient electroporation protocol for gene editing in primary human cells using the Cas9 RNP complex. Supplementary Table 4 shows the steps taken to develop this improved protocol, which is now available and can be reproduced. Using this protocol, we achieved, on average, a relatively high yield of gene disruption (51%) that significantly affected cell function. Together with previous reports^49,50^, we show that ex vivo electroporation-based gene editing is safe and effective.

### Limitations

We are aware of several limitations of our work. First, our protocol created a heterogeneous population of edited and unedited cells. Second, although edited hMSCs improved LV remodeling and function, many favorable effects were similar to unedited hMSCs. The absence of significant functional advantage for edited over unedited cells may be related to the heterogeneity of the edited-cell group and the fact that nearly 50% of the cells remained unedited. A longer follow-up period may reveal significant differences between the edited and unedited cells. Still, the effects on transmural infarcts, infarct expansion, and myocardial islands were more substantial with edited-hMSC therapy.

Another limitation of this study is the ECM composition. Collagen expression in the edited hMSCs’ conditioned medium was downregulated. We did not analyze sub-types of collagen in the infarcted hearts because we, and others, believe that resident hMSCs/fibroblasts are the major sources of collagen in the infarcted tissue^51–55^. Resident fibroblasts may be affected by implanted cells, either directly or via modulation of inflammation^56^.

Furthermore, we believe that late injection may yield different results. However, due to the high mortality associated with a second thoracotomy in mice and difficulties related to the delivery of cells into the thin (< 0.5 mm) scar of a mouse within days after MI, we injected the cells immediately after MI. Another aspect of cell transplantation that can benefit from further research is the change in the level of DAMPs released from resident cells. We did not measure DAMPs following the transplantation of hMSCs. Although DAMP release after MI is well documented^57^, it is unknown whether hMSC transplantation affects levels of DAMPs. hMSC transplantation early after MI may modulate the levels of DAMPs. Still, the immune response and modulation following MSC transplantation have both been extensively described by us and others^58–63^. The immune response is highly dependent on the source of cells (healthy or sick tissues)^58–63^.

In addition, in this study, we focused on LV remodeling and scar formation, and did not assess angiogenesis in the infracted and remote myocardial tissue. As a result, we found conflicting results: while cell editing resulted in a modest downregulation of VEGF secretion, adding conditioned medium from edited hMSCs stimulated endothelial cell tube formation (Fig. 4C). A possible explanation for this is the upregulation of other unidentified angiogenic factors or downregulation of angiogenesis inhibitors such as IL-10^64^ or sFLT1 (we did not measure this in the present experiment).

Finally, although we implanted human cells in mice, we did not use immunosuppression. The rationale for avoiding immunosuppression was that acute immune response and immunomodulation play central roles in improving LV remodeling and function by MSCs^56,61^. Therefore, we avoided immunosuppression. Consequently, an immune response against the implanted human cells may compromise hMSC survival. Indeed, we noticed inflammatory infiltrates at the site of cell implantation, particularly around unedited hMSCs. Contrary to unedited cells, edited cells showed high resilience and survival of human cells in the mouse tissue. However, the survival of implanted MSCs in the infarcted myocardium is limited even with the syngeneic model of cell therapy^12^.

## Summary

We combined the power of genome editing and cell-based therapy to engineer better cells for heart repair (Fig. 7). CRISPR-based disruption of the TLR4 gene in hMSCs facilitates a reparative response in vitro and in vivo. The ex vivo approach to cell editing may generate new, function-modified, personalized cells for heart repair. Autologous edited cells may be relevant in treating elderly patients with massive myocardial damage, as well as patients whose myogenic or angiogenic cells have been depleted or have inadequate reparative potential to prevent LV deterioration and heart failure. A better understanding of molecular and cellular mechanisms underlying heart repair is needed to improve gene editing, efficacy, and safety, identify relevant targets, and create better cells for heart repair.

## Methods

A detailed description of the methods is provided in the online Supplemental Materials. In addition, all data that support the findings are available within the article, in the Supplemental Materials, or upon reasonable request.

### Patients, sample collection, and cell isolation

An institutional Helsinki review board approved the study at Sheba Medical Center and Tel Aviv University. The participants gave written informed consent. All comply with the Declaration of Helsinki.

We obtained samples of tissues from the right atrial appendage and epicardial fat of patients undergoing elective open-heart surgery. We isolated and grew the human cardiac mesenchymal stromal cells (hMSCs), as previously described^9^.

### Ribonucleoprotein (RNP) complex

We used recombinant Cas9 nucleases, synthetic CRISPR RNA (crRNA), and trans-activating CRISPR RNA (tracrRNA) (Alt-R S.p. Cas9 Nuclease V3, IDT, Coralville, IA, USA). crRNA was designed according to the Benchling Guide RNA Design Tool (https://benchling.com). crRNA and tracrRNA were annealed according to the manufacturer’s instructions. Control groups received non-specific scrambled single guide RNA (sgRNA) (IDT, Coralville, IA, USA).

### Electroporation-based Crispr-Cas9 gene editing

The electroporation setup contained: RNP complex, hMSC electroporation enhancer by IDT, and DMEM medium. A single square wave pulse of 125 V for 5 ms was applied once using an ECM 830 (BTX, Cambridge, UK).

### Sequencing and analyzing results

To determine the efficacy of the gene-editing reaction, we processed and examined the cells for insertion or deletion (indel) rate two days after electroporation. Processing involved the Genomic DNA Extraction Kit (Invitrogen, Carlsbad, CA, USA) and the Platinum SuperFi PCR Master Mix (Invitrogen, Carlsbad, CA, USA) with the Benchling Primer Design Tool^65^ used for primer design, all according to the manufacturer’s instructions. PCR products were validated with a 2% agarose gel and sent to Macrogen (Amsterdam, Netherlands) for Sanger sequencing. Cell sequencing results were analyzed using the tracking of indels by decomposition (TIDE) assay (https://tide.nki.nl)^20^.

### Flow cytometry

We used flow cytometry to validate hMSC surface markers on edited and unedited cells. Cluster of differentiation (CD)90, CD105, CD73, CD34, CD45, and TLR4 (BioLegend, San Diego, CA) were used according to the manufacturer's instructions.

### Cell proliferation colorimetric assay

To determine the effect of TLR4 disruption in hMSCs on cell number and growth, we used the Cell Proliferation Kit (2,3-bis-(2-methoxy-4-nitro-5-sulfophenyl)-2H-tetrazolium-5-carboxanilide, XTT) (Biological Industries, Beit HaEmek, Israel) according to the manufacturer's protocol.

### Fibroblast migration scratch assay

To determine the effect of TLR4 gene disruption on cell migration, we used a fibroblast migration ("scratch") assay^66^. In short, we used a 10 µL tip to scratch a well of 90% confluence. We then measured the gap between the marginal of the cells every hour until the cells closed the gap.

### hMSC cell culture staining

To stain cultured hMSCs for various markers, we washed, fixed, and immunostained the cells with specific antibodies against their isotype control. (A full list of antibodies is available in the Supplemental Materials.)

### TLR4 marker by western blot

Anti-human TLR4 marker for hMSCs was analyzed by western blot (bs-20594R, Bioss Antibodies, MA, USA).

### RNA extraction and quantitative reverse transcription PCR (qRT-PCR)

We performed TLR4 gene expression analysis in cultured hMSCs using qRT-PCR. Total RNA was extracted from hMSCs using the RNeasy Mini Kit (Qiagen, Germantown, MD. USA), cDNA library prep, and TaqMan assays (Applied Biosystems, Waltham, MA, USA) were performed according to the manufacturer’s instructions. 2^−ΔΔCt^ values were normalized to GAPDH.

### Purification of EVs by size exclusion chromatography (SEC)

Our methods for the isolation of epicardial fat-derived EVs were guided by the recent position statement of the International Society for Extracellular Vesicles (MISEV2018)^67^. Accordingly, we isolated EVs by Izon qEV columns (IZON, Oxford, UK)^68^.

### Nanoparticle tracking analysis (NTA)

To measure the amount and size distribution of isolated EVs, we used NTA using the Malvern NanoSight NS300 (Malvern, Grovewood Road, UK).

### Proteolysis and mass spectrometry analysis

To evaluate proteins secreted from hMSCs directly in conditioned media and indirectly by EVs, we performed Proteomic analysis. We isolated EVs by size exclusion chromatography column (SEC) and analyzed them with a Q-Exactive Plus mass spectrometer (Thermo Fisher Scientific, Waltham, MA, USA). In addition, we analyzed pathway enrichments with STRING using the PANTHER Pathway keywords. We deposited mass spectrometry proteomics data to the ProteomeXchange Consortium via the PRIDE^69^ partner repository with the dataset identifier PXD033253.

### Cytokine array

To determine the effect of gene editing on cytokine secretion, we used the Q-Plex Human Cytokine Array, 4-Plex, and a custom plate (Quansys Biosciences Multiplex ELISA, West Logan, UT, USA)^70^. Concentrations of cytokines were determined by Quasys Q-View imaging and a software system.

### Angiogenic tube formation assay

To determine the angiogenic properties of the edited-cell conditioned medium, we used the Human Dermal Microvascular Endothelial Cells (HDMEC) Matrigel Tube Formation Assay^71^. The number of formed tubes was evaluated after 3.5 h.

### Animal care

This study was performed under the guidelines of the Animal Care and Use Committee of the Sheba Medical Center. This study complies with the ARRIVE guidelines.

### Myocardial infarction in adult mice

To determine the impact of cell therapy on infarct repair, we used a mouse model of MI in 12-week-old female Balb/C mice (Harlan Laboratories, Jerusalem, Israel), as previously described^9,56^. MI was confirmed by immediate visual blanching and wall motion akinesis distal to the occlusion site, and by echocardiography 24 h after MI.

### Cell therapy

We used our previously reported protocol to deliver hMSCs to the infarcted heart^10^. Mice were allocated to three experimental groups, using a color code for double-blinding purposes: one control group with Dulbecco's phosphate buffered saline (PBS) and two experimental groups with edited or unedited cells. 100,000 cells in 20 µL of PBS were washed 3 times. Cells were kept on ice until 5 min before use. Then, mice were treated with a single injection of cell therapy to the border of the ischemic zone one minute after coronary artery ligation. The chest was sutured, and mice were placed on a heating pad (37 °C) until recovery.

### Echocardiography to evaluate cardiac function

To assess LV remodeling and function after myocardial injury in mice, we used a small animal echocardiography system (Vevo 2100 Imaging System; VisualSonics, Toronto, Ontario, Canada) equipped with a 22- to 55-MHz linear-array transducer (MS550D MicroScan Transducer). Echocardiographic studies were performed before surgery and on days 1, 8, and 28 after injury and treatment.

### Histologic analysis

To assess myocardial injury, healing, repair, and regeneration after MI and cell engraftment, we harvested the hearts, washed them, and then fixed them on day 29 after MI. Adjacent blocks were embedded in paraffin, sectioned, and stained for further analysis.

### Statistical analysis

Statistical analyses were performed using GraphPad Prism 9.2 for Windows (GraphPad Software, San Diego, California USA, www.graphpad.com). Variables are expressed as mean ± standard deviation (SD). Statistical tests are detailed in the figure legends and in the Supplementary Methods.

## Supplementary Information

Supplementary Information 1.

Supplementary Information 2.

Supplementary Information 3.

## Data Availability

The authors declare that all other data supporting the findings of this study are available within the paper (and its supplementary information files). Additionally, the datasets generated and/or analyzed during the current study are available in the ProteomeXchange Consortium via the PRIDE^69^ partner repository with the dataset identifier PXD033253.
