# Supplementary material for: CRISPR-Cas9 editing of TLR4 to improve the outcome of cardiac cell therapy
Source: Sci Rep. 2023 Mar 18;13:4481. doi: 10.1038/s41598-023-31286-4 (PMC10024743; doi:10.1038/s41598-023-31286-4)
Supplement: Supplementary file 1 — Supplementary Information 1. [file 41598_2023_31286_MOESM1_ESM.pdf]

CRISPR-Cas9 Editing of *TLR4* to Improve the Outcome of Cardiac Cell Therapy

Supplemental Material

Yeshai Schary, MSc[1,2]; Itai Rotem, MSc[1,2]; Tal Caller, BMedSc[1,2]; Nir Lewis, PhD[1,2]; Olga Shaihov-Teper, MSc[1,2]; Rafael Y. Brzezinski, PhD[1,2]; Daria Lendengolts, DVM[1,2]; Ehud Raanani, MD[2,3]; Leonid Sternik, MD[2,3]; Nili Naftali-Shani, PhD[1,2] and *Jonathan Leor, MD[1,2].

[1]Neufeld and Tamman Cardiovascular Research Institutes, Sackler School of Medicine, Tel Aviv University, Israel

[2]Heart Center, Sheba Medical Center, Tel Hashomer, Israel

[3]Department of Cardiac Surgery, Leviev Cardiothoracic and Vascular Center, Sheba Medical Center, Tel Aviv University, Israel

***Corresponding author**:
Jonathan Leor, MD, FACC, Neufeld Cardiac Research Institute,
Sheba Medical Center, Tel-Hashomer 52621, Israel.
Tel: +972-52-666-7112, +972-3-530-2614. Fax: +972-3-535-1139.;
E-mail: leorj@tauex.tau.ac.il. Twitter: @LEORJONATHAN

Supplement Figure 1

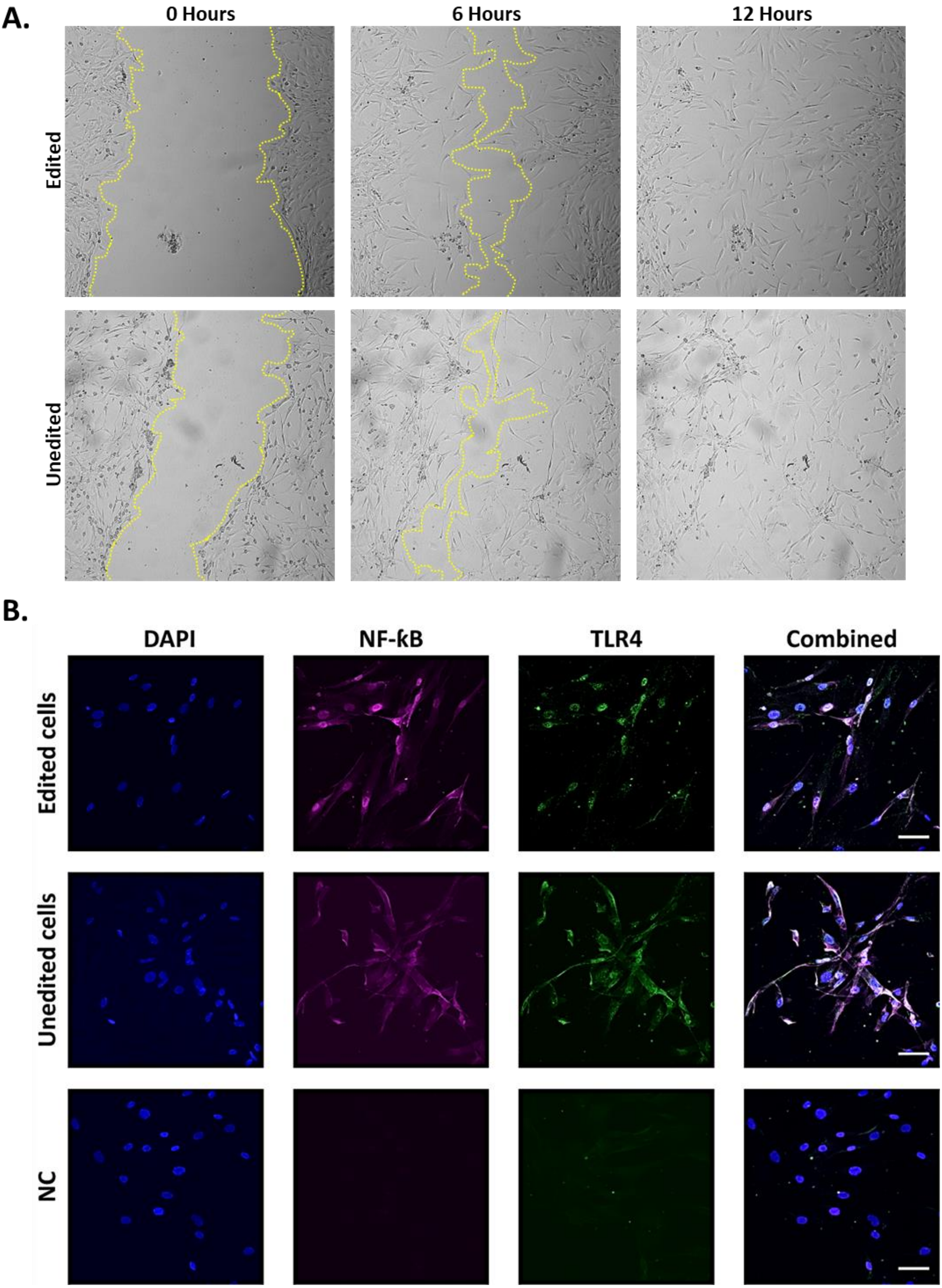

SUPPLEMENTARY FIGURE 1

Cell in vitro assays

(A) Representative images of hMSCs at three-time points. Each sample is a mean of technical triplicates. Cells were seeded to full confluence in a 96-well plate. Each scratch was performed using a fresh 10 µL tip, the gap area marked manually and calculated using ImageJ.

(B) Representative immunofluorescent images of hMSCs with anti-TLR4 and anti-NF-ƙB antibodies (confocal microscopy using LSM 700, ZEISS).

Supplement Figure 2

A.

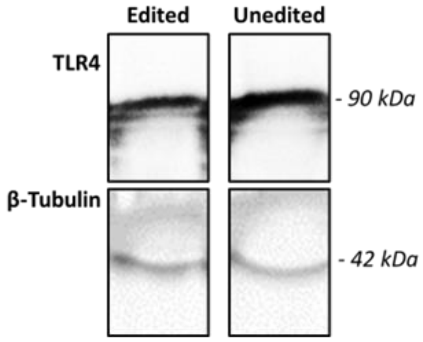

B.

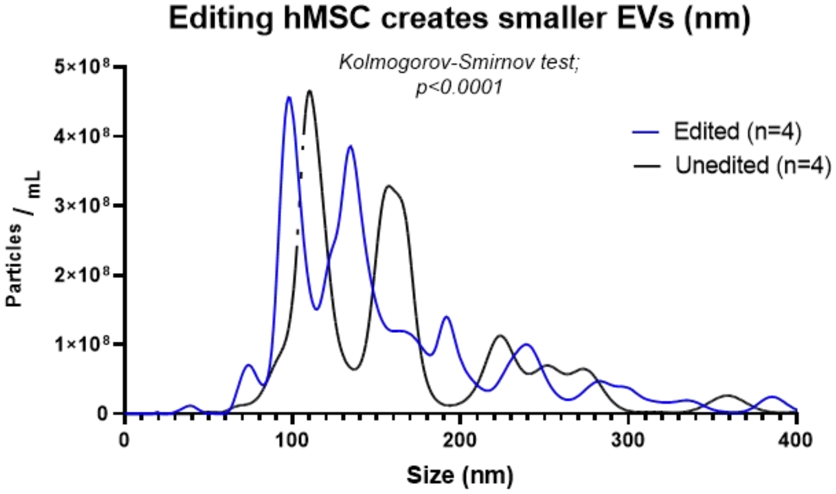

C.

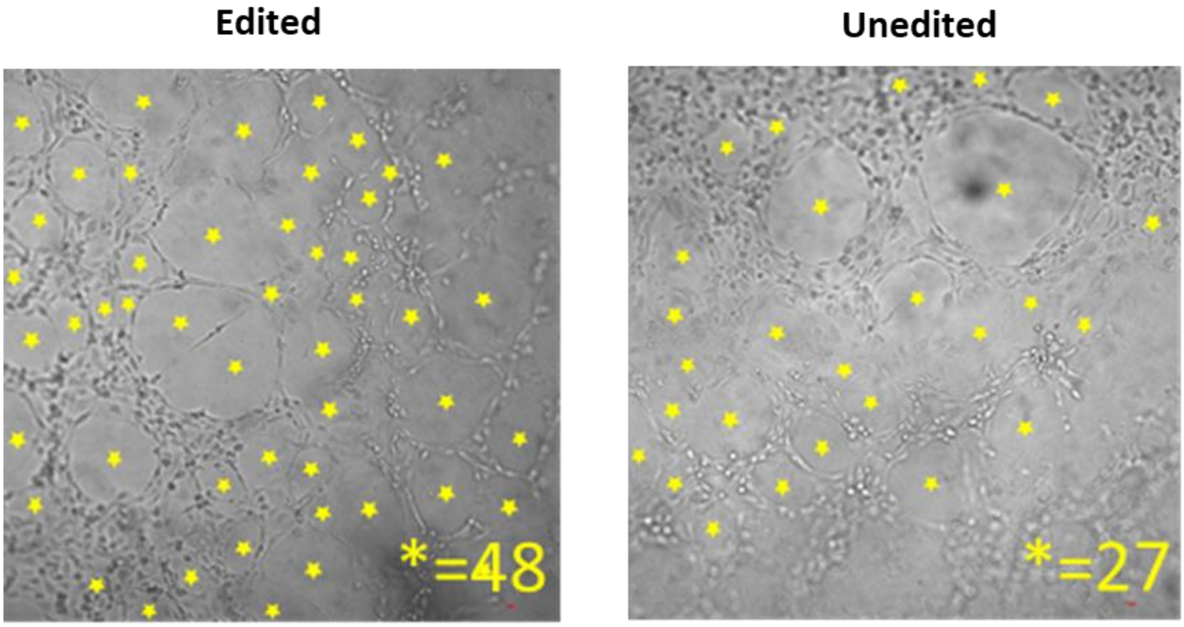

SUPPLEMENTARY FIGURE 2

Cell reaction to gene editing

(**A**) Original protein-gel bands analyzed with the ImageJ Gel Analysis plugin normalized to the expression level of the housekeeping gene for β-tubulin.

(**B**) EV distribution concentration found using nanoparticle tracking analysis (NTA) of EVs isolated from $1x10^{6}$ cells in no-FBS growth medium, incubated for three consecutive days. Each sample is a mean of distribution from technical triplicates. EVs were purified by size-exclusion chromatography (SEC).

(**C**) Representative images of human dermal microvascular endothelial cells (HDMEC) incubated with conditioned growth media combined with HDMEC media, images analyzed 3.5 hours after start time. Each sample is a mean of three triplicates.

SUPPLEMENTARY METHODS

The authors declare that all supporting data are available within the article and its online supplementary files.

The data, methods used in the analysis, and materials used to conduct the research available to any researcher from the corresponding author upon reasonable request.

Patients, Sample Collection and Cell Isolation

The study was approved by the institutional review boards of the Sheba Medical Centre and Tel Aviv University. The participants gave written informed consent. For this study, samples of 4 patients were randomly chosen. Samples of human epicardial fat heart tissue were obtained from patients undergoing elective open-heart surgery (***Supplementary Table 2***). We excised small samples with a surgical scalpel to avoid thermal injury to the tissue. Human mesenchymal stromal cells (hMSCs) were extracted with an enzymatic digestion mixture. Adhered cells were grown and expanded as previously described.[53]

Ribonucleoprotein (RNP) Complex

We purchased recombinant Cas9 nucleases (Alt-R® S.p. Cas9 Nuclease V3, IDT, Coralville, IA, USA) as well as synthetic crRNA and tracrRNA from the same supplier. crRNA was designed according to Benchling guide-RNA design tool (https://benchling.com/). Three crRNAs were targeted at exon I

(ATAGCGAGCCACGCATTCAC, GATGATGTCTGCCTCGCGCC, TCAGAAACTGCTCGGTCAGA) and one crRNA at exon III on the revers DNA strand of the TLR4 gene in the human genome (GATGCCATTGAAAGCAACTC). In this study the fourth crRNA mentioned was used. crRNA:tracrRNA was annealed to a single guide RNA (sgRNA) of 30 pmol/µl and then complexed with a 20 pmol/µl recombinant Cas9 at a ratio of 3:1 to form a final concentration of 20 pmol/µl RNP complex (10 minutes at room temperature), all according to the manufacturer's manual. Negative control groups where all electroporated with non-specific scrambled sgRNA (IDT, Coralville, IA, USA).

Electroporation Protocol

To insert CCRISPR-Cas9 into hMSCs, we used electroporation. The reaction setup is prepared in a 1-mm cuvette that contains (amount, final concentration): (1) RNP complex (3 µL, 0.75 µM). (2) Electroporation enhancer by IDT (2 µL, 2.5 µM). (3) DMEM medium (15 µL). (4) Human cardiac mesenchymal cells (hMSCs) (60 µL, $1x10^6$). Total volume of reaction: 80 µL. The loading order in the cuvettes was set for best mixing: RNP, electroporation enhancer, DMEM, and finally the cells. A single square wave pulse of 125 V for 5 ms was applied once using an ECM 830 (BTX, Cambridge, UK). After electroporation, the cells rested for 1-2 minutes and were then transferred to a 75-mL flask with pre-warmed growth medium using a thin sterile disposable 1 mL soft-plastic pipette for each reaction. Immediately after, cells were placed in the incubator. 24 hours after initial seeding, cells where washed and growth medium replenished to dispose of debris from dead cells.

Sequencing and Analyzing Results

To evaluate the efficacy of the gene editing reaction, cells were examined for indel-rate two days after electroporation by extraction of genomic DNA (gDNA). 50,000-250,000 cells were extracted from the culture and processed with a genomic DNA extraction kit (Invitrogen, Carlsbad, CA, USA) according to the manufacturer's protocol. DNA sequence spanning the sgRNA target was amplified with PCR (FWD – TGGGACAACCAGCCTAAAGTAT, REV – ACCTGAAGACTGGAGAGTGAGT) using Platinum SuperFi PCR Master Mix (Invitrogen, Carlsbad, CA, USA) with primers planned using the Benchling Primer Design Tool. PCR products were validated in a 2% agarose gel to have a 384-bp band and PCR products were sent to sequence analysis (Macrogen, Amsterdam, Netherlands). Cell sequencing results were analyzed using TIDE (https://tide.nki.nl/). Edited cells used in this study had > 50% indel rate.

Flow Cytometry

Flow cytometry was used to validate markers on edited and unedited MSCs. Specific antibodies for CD90, CD105, CD73, CD 34, CD45, and TLR4 were purchased from BioLegend (San Diego, CA). Analysis was performed according to manufacturer's protocol briefly: $0.5x10^6$ cells were harvested and stained with the specific antibody for 0.5 hours at room temperature. Cells were then washed three times and resuspended in 400 µl PBS. All measurements were read with 100,000 cells and performed by flow cytometry using a FACSCalibur flow cytometer (Cyteck

Development, Fremont, CA, USA) and analysed by FlowJo 10.1 (Tree Star, Ashland, OR, USA). Gating of each antibody was performed with a matching isotype control.

hMSC Culture staining

To stain cultured hMSCs for various markers, we washed and fixed the cells using 4% formalin. Membrane permeability was achieved with Triton 0.2% X-100 for one minute at room temperature followed by blocking for non-specific sites using CAS-Block (008120, Life Technologies, Freseric, MD, USA) for 10 min. Next, primary antibodies were added against hTLR4 (1:00, ab22048, abcam, Cambridge, UK) and hNF-ƙB (1:200, ab16502, Abcam, Cambridge, UK), and incubated for two hours at 4°C. Then, the secondary antibodies Alexa Fluor 488 (1:200, Jackson ImmunoResearch, Cambridge, UK), Texas Red (1:200, Jackson ImmunoResearch, Cambridge, UK), and Mounting Gel with DAPI (Bar-Naor, Petah Tikva, Israel) were added.

Angiogenic Tube Formation Assay

To determine the angiogenic properties of edited-cell-preconditioned medium we used a human dermal microvascular endothelial cell (HDMEC) Matrigel tube formation assay. HDMECs (Promocell, Biological Industries, Beit HaEmek, Israel) were seeded at a concentration of $3x10^4$ cell/well in a 96-well plate pre-coated with 50 µL Matrigel matrix (BD Biosciences, San Jose, CA, USA) and allowed to attach. Next, the HDMECs were incubated with 50 µL of HDMEC growth media

(manufacturer's protocol) and 50 µL of pre-conditioned growth media from edited and unedited cells. Images of each well were automatically taken every 30 minutes in each well for six hours with a laser scanning confocal microscope LSM 700 (Zeiss, Oberkochen, Germany). The number of formed tubes was evaluated after 3.5 hours.

Cytokine Array

To determine the effect of cell editing on cytokine secretion we used Q-Plex^{TM} Human Cytokine (4-Plex and custom plate) array (Quansys Biosciences Multiplex ELISA, West Logan, UT, USA), according to the manufacturer's instructions. Multiplex ELISA was performed on a sample from the right atrial appendage. The array was designed to quantitatively detect all or some of the following cytokines: IFN-γ, IL-1α, IL-1β, IL-2, IL-6, IL-8, IL-10, and VEGF. Briefly, we cultured 50,000 hMSCs for 72 hours in a 24-well plate and collected the secreted medium. To determine the cytokine concentration in each sample, an image of the plate was acquired by the Quansys Q-View imaging system, which includes a high-resolution Canon digital camera. The intensity of the chemiluminescence of each spot was compared with that of an 8-point standard curve generated by 5-parameter logistic regression for that particular cytokine, using Quansys Q-View software.

Western blot

hMSCs were analyzed for TLR4 protein synthesis by western blot, probed for human TL4 (bs-20594R, Bioss ANTIBODIES, MA, USA). Appropriate secondary antibody was

used (Thermo-Fisher Scientific, Waltham, MA, USA), and detection was carried out using the enhanced chemiluminescent reagent WESTAR NOVA 2.0 (CYANAGEN, Bologna, Italy). Before the procedure, isolated hMSCs were treated with RIPA buffer (Thermo-Fisher Scientific, Waltham, MA, USA) to expose their content.

RNA Extraction and Quantitative Reverse Transcription PCR (qRT-PCR)

Gene expression analysis in cultured MSCs was performed by qRT-PCR. Total RNA was extracted from hMSCs using the RNeasy Mini Kit (Qiagen, Germantown, MD, USA), according to the manufacturer's protocol. The cDNA was generated from total RNA using the High-Capacity cDNA RT Kit (Qiagen, Germantown, MD, USA). qRT-PCR was performed in triplicates using human TLR4 and GAPDH TaqMan Gene Expression Assays with TaqMan Fast Advanced Master Mix (Applied Biosystems, Waltham, MA, USA) according to the manufacturer's protocol. $2^{-\Delta\Delta Ct}$ values were normalized to GAPDH.

XTT Colorimetric Assay

To determine the effect of TLR4 KO in hMSCs on cell number and growth, we used Cell Proliferation Kit (XTT-based) (Biological Industries, Beit HaEmek, Israel). $5x10^4$ cells were seeded in a 96-well plate, and after 24 hours cells were washed once and 100 µL of fresh growth medium added. The Proliferation assay was performed according to the manufacturer's protocol. Every day a technical triplicate for each sample of conditioned medium was incubated for two hours in the dark at 37°C, and

then light absorbance was measured at 492 nm wavelength with a reference read at 620 nm in a spectrophotometer.

Fibroblast Migration Scratch Assay

To demonstrate a causative association between TLR4 KO to anti-inflammatory properties we used a fibroblast migration ("scratch") assay. Unedited primary hMSCs were seeded at a density of $1x10^4$ cells in a 96-well plate and grown to 90% confluence. Grown cells were scratched with a sterile 10 µL pipette tip. After washing cells three times, 100 µL of fresh, serum-free preconditioned culture medium from $1x10^6$ cells were added. Images of the scratched area were automatically taken every 60 minutes in each well for 24 hours with the laser scanning confocal microscope LSM 700 (Zeiss, Oberkochen, Germany). The scratched area was measured by ImageJ FIJI 1.52p (Wayne Rasband, NIH, Bethesda, MD, USA).[54]

Purification of EVs by Size Exclusion Chromatography (SEC)

Our methods for isolating hMSCs-derived EVs were guided by the recent position statement of the International Society for Extracellular Vesicles (MISEV2018).[55] EVs were isolated by Izon qEV columns (IZON, Oxford, UK) according to the manufacturer's protocol and as previously described by our group.[56] Briefly, conditioned medium was centrifuged at 1,500 g for 10 min using a Heraeus Labofuge 400 Centrifuge (Thermo-Fisher Scientific, Waltham, MA, USA) to remove any cells

and large particles. The supernatant was collected and centrifuged at 10,000xg at 4°C for 10 min, using a Sorvall LYNX 6000 Superspeed Centrifuge (Thermo-Fisher Scientific, Waltham, MA, USA). The supernatant was collected and then loaded onto the loading frit of the column. The sample was allowed to run into the column, and the eluate was collected in 36 sequential fractions of 2 mL. Next, we concentrated the EVs by short ultracentrifugation. Briefly, the main pooled fractions were centrifuged at 100,000xg (40,833 rpm) at 4°C for 70 min in 5/8 x 3 in. (16 x 76mm) Quick-Seal polypropylene centrifuge tubes (BECKMAN COULTER, Pasadena, CA, USA) using a type 50 Ti fixed-angle rotor, r_{av}=59.1, k-factor=97.1, using a Sorval WX Ultracentrifuge (Thermo-Fisher Scientific, Waltham, MA, USA). The supernatant was discarded, and the EV pellet was suspended in PBS, divided into aliquots, and stored at -80°C for further use.

Nanoparticle Tracking Analysis (NTA)

The size distribution of isolated EVs was examined by the Malvern NanoSight NS300 (Malvern, UK). Each particle in the medium was simultaneously analyzed for both particle size and concentration. The samples were diluted 1:700 in sterile-filtered PBS and analyzed. The measurements were based on three one-minute-long videos, with camera level 11, detection threshold 2, and screen gain 10. Results were analyzed using the NTA software package 3.00.

Proteolysis and Mass Spectrometry Analysis

The mass spectrometry proteomics data have been deposited to the ProteomeXchange Consortium via the PRIDE[57] partner repository with the dataset identifier PXD033253.

Reviewer account details:

Username: reviewer_pxd033253@ebi.ac.uk

Password: jwawlxxe

Proteomic analysis was done as previously described.[56] In short, samples of EVs, isolated by SEC, were brought to 10 mM DTT, 100 mM Tris, and 4% SDS, boiled at 95°C for 5 min and sonicated. The samples were precipitated in 80% acetone. The protein pellets were dissolved in 9 M urea, 400 mM ammonium bicarbonate, 10 mM DTT, then reduced to 60°C for 30 min, modified with 40 mM iodoacetamide in 100 mM ammonium bicarbonate (at room temperature for 30 min in the dark) and digested in 1.5 M urea, 66 mM ammonium bicarbonate with modified trypsin (Promega, Madison, WI, USA), overnight at 37°C in a 1:50 (M/M) enzyme-to-substrate ratio. Additional second trypsinization was performed for 4 hours.

The tryptic peptides were desalted using C18 tips (TopTip, Glygen) dried and resuspended in 0.1% formic acid. The peptides were resolved by reverse-phase chromatography on 0.075 x 200-mm fused silica capillaries (J&W) packed with Reprosil reversed-phase material (Dr. Maisch GmbH, Germany). The peptides were eluted with a linear 105-minute gradient of 5% to 28% acetonitrile, with 0.1% formic acid in the water, a 15-minute gradient of 28% to 90% acetonitrile with 0.1% formic acid in water, and 15 minutes at 90% acetonitrile, with 0.1% formic acid in water at

flow rates of 0.15 µl/min. Mass spectrometry (MS) was performed by a Q-Exactive Plus mass spectrometer (QE, Thermo-Fisher, Waltham, MA, USA) in a positive mode using a repetitively full MS scan followed by high energy collision dissociation of the ten most dominant ions selected from the first MS scan. Samples ran in duplicates.

Data from the MS were analyzed by MaxQuant software 1.5.2.8. (https://www.maxquant.org/) using the Andromeda search engine, searching against the human proteome from the UniProt database with a mass tolerance of 20 ppm for the precursor masses and 20 ppm for the fragment ions. Peptide- and protein-level false discovery rates (FDRs) were filtered to 1% using the target-decoy strategy. A protein table was filtered to eliminate the identifications from the reverse database, common contaminants, and single peptide identifications. Data were quantified by label-free analysis using the same software, based on extracted ion currents (XICs) of peptides, enabling quantitation from each LC/MS run for each peptide identified in each experiment.

Proteins were clustered in categories depending on their known main biological function using the open-source bioinformatics resource STRING[58] Protein-Protein Interaction Networks Functional Enrichment Analysis (https://string-db.org). The homo-sapiens genome was employed as a background list. Pathway enrichment analysis was performed with STRING using the PANTHER pathway keywords and exported as a bar chart of representation number of proteins.

Statistical analysis of the identification and quantization results was performed using Perseus 1.6.7.0 (Mathias Mann's group, Max Planck Institute of Biochemistry, Martinsried, Germany).

Myocardial Infarction in Adult Mice

This study has been performed in accordance with the guidelines of the Animal Care and Use Committee of the Sheba Medical Center.

To determine the impact of injecting edited cells on infarct repair, we used a mouse model of MI previously described.[53,59] In short, we used 10-12-week-old Balb/C female mice (Harlan Laboratories, Jerusalem, Israel). Mice were anesthetized with 2-2.5% isoflurane, intubated, and ventilated with 100% oxygen. The chest was shaved and opened by left thoracotomy, and coronary artery ligation was performed using an 8-0 prolene suture (Ethicon, Cornelia, GA, USA) to permanently occlude the left anterior descending coronary artery. Immediately after the occlusion, cells were injected to the infarcted area. Myocardial ischemia was confirmed by visual blanching distal to the occlusion site, with wall-motion akinesis. We also confirmed MI by echocardiography 24 hours after surgery. The chest was closed, and the skin glued with a biological glue (Histoacryl, B. Braun Surgical S.A, Rubi, Spain). Mice were placed under a warming light until recovery. Survival rate after MI for the control group with PBS was 70%, and the average survival rate with cell treatments was 95%.

Cell Therapy

To determine the reparative effects of edited cells, we used a protocol of cell therapy in a mouse model of MI.[60] Mice (Balb/C females, 12-week-old) were

allocated to three experimental groups using a color code for double blinding purposes. Negative control was treated with PBS, and two experimental groups with edited or unedited cells.

On the morning of the experiment, cells (80% confluency) were counted, washed three times with PBS at 4^oC to remove serum residuals from the cells, and concentrated to 100,000 cells in 20 µL of PBS. Following that, exactly 20 µL of the cells (edited or unedited) or saline were aspirated into a 1 mL insulin syringe. All materials were kept on ice until 5 minutes before use.

We did not use immunosuppression. The rationale for avoiding immunosuppression was the critical role of local immune response and immunomodulation in the mechanism by which the implanted cells improve infarct repair, LV remodeling and function.[61,62] Thus, we did not use immunosuppression.

Echocardiography to Evaluate Cardiac Function

To assess LV remodeling and function after myocardial injury in mice, we used a special small animal echocardiography system (Vevo 2100 Imaging System; VisualSonics, Toronto, Ontario, Canada) equipped with a 22- to 55-MHz linear-array transducer (MS550D MicroScan Transducer; VisualSonics, Toronto, Ontario, Canada). Echocardiographic studies were performed at baseline, and at days 1, 8, and 28 after injury and treatment. Light anesthesia was induced by inhalation of 2-2.5% isoflurane/98% O_2 and subsequently maintained by 2-2.5% isoflurane. We controlled the isoflurane flow to maintain a heart rate >400 bpm. All measurements were

averaged over three consecutive cardiac cycles and performed by an experienced technician blinded to the treatment groups. Values were calculated for each animal as follows: Ejection fraction = [(LV vol d-LV vol s)/LV vol d]×100; Fractional shortening = [(LVDD−LVSD)/LVDD]×100. Exclusion criteria for mice were EF<40% on baseline echo or EF>40% on day 1 echo.

Histologic Analysis

To assess myocardial injury, healing, and repair after MI, hearts were harvested at day 29 after the procedure, washed with PBS and then fixed in 4% paraformaldehyde overnight. Adjacent blocks were embedded in paraffin and sectioned into 5 µm slices. Hematoxylin and eosin, and picrosirius red staining (to detect scarring and fibrosis) were performed according to standard procedure.

Postmortem morphometric analysis

Postmortem morphometric analysis was performed on hearts from the adult MI experiment, as previously described [59]. The slides were stained with hematoxylin and eosin and picrosirius red, photographed and analyzed with planimetry software (Sigma Scan Pro 5, Systat Software, San Jose, California, USA). We measured LV maximal diameter defined as the longest diameter perpendicular to a line connecting the insertions of the septum to the ventricular wall, average wall thickness from 3 measurements of septum thickness, average scar thickness from 3 measurements of scar thickness, LV muscle area (including the septum), LV cavity

area, whole LV area, epicardial scar length (millimeters), and endocardial scar length (millimeters). Relative scar thickness was calculated as average scar thickness divided by average wall thickness. Expansion index was calculated as follows: [LV cavity area/whole LV area]/relative scar thickness].

Statistical Analyses

Statistical analyses were performed with GraphPad Prism 9.1.1 (GraphPad Software). Experimental data are expressed as mean ± standard error of the mean (SEM). Specific statistical tests are detailed in the figure legends. In brief, the differences between groups in the experimental data were tested by unpaired t-test or one-way analysis of variance (ANOVA) (>2 groups). If values were not normally distributed (tested by the D'Agostino-Pearson omnibus normality test), then we used the nonparametric Mann-Whitney test or Kruskal-Wallis test (>2 groups), followed by Dunn's multiple comparisons post-test. To assess the correlation between free cytokines and EV encapsulated cytokines, we used Spearman's correlation test. To assess the significance of predefined comparisons at specific time points, we used two-way repeated-measures analysis of variance (ANOVA) with Holm-Šídák's multiple comparisons post-test. To avoid bias, mice that died after day 1 measurements were excluded from the experiment.

REFERENCES

53. Naftali-Shani N, Itzhaki-Alfia A, Landa-Rouben N, Kain D, Holbova R, Adutler-Lieber S, Molotski N, Asher E, Grupper A, Millet E, et al. The origin of human

mesenchymal stromal cells dictates their reparative properties. *J Am Heart Assoc*. 2013;2:e000253. doi: 10.1161/JAHA.113.000253
54. Schindelin J, Arganda-Carreras I, Frise E, Kaynig V, Longair M, Pietzsch T, Preibisch S, Rueden C, Saalfeld S, Schmid B, et al. Fiji: an open-source platform for biological-image analysis. *Nat Methods*. 2012;9:676-682. doi: 10.1038/nmeth.2019
55. Thery C, Witwer KW, Aikawa E, Alcaraz MJ, Anderson JD, Andriantsitohaina R, at. e. Minimal information for studies of extracellular vesicles 2018 (MISEV2018): a position statement of the International Society for Extracellular Vesicles and update of the MISEV2014 guidelines. *J Extracell Vesicles*. 2018;7:1535750. doi: 10.1080/20013078.2018.1535750
56. Shaihov-Teper O, Ram E, Ballan N, Brzezinski RY, Naftali-Shani N, Masoud R, Ziv T, Lewis N, Schary Y, Levin-Kotler L-P, et al. Extracellular Vesicles from Epicardial Fat Facilitate Atrial Fibrillation. *Circulation*. 2021. doi: 10.1161/CIRCULATIONAHA.120.052009
57. Perez-Riverol Y, Bai J, Bandla C, García-Seisdedos D, Hewapathirana S, Kamatchinathan S, Deepti, Prakash A, Frericks-Zipper A, Eisenacher M, et al. The PRIDE database resources in 2022: a hub for mass spectrometry-based proteomics evidences. *Nucleic Acids Research*. 2022;50:D543-D552. doi: 10.1093/nar/gkab1038
58. Szklarczyk D, Gable AL, Nastou KC, Lyon D, Kirsch R, Pyysalo S, Doncheva NT, Legeay M, Fang T, Bork P, et al. The STRING database in 2021: customizable protein-protein networks, and functional characterization of user-uploaded gene/measurement sets. *Nucleic acids research*. 2021;49:D605-D612. doi: 10.1093/NAR/GKAA1074
59. Landa N, Miller L, Feinberg MS, Holbova R, Shachar M, Freeman I, Cohen S, Leor J. Effect of injectable alginate implant on cardiac remodeling and function after recent and old infarcts in rat. *Circulation*. 2008;117:1388-1396. doi: 10.1161/CIRCULATIONAHA.107.727420
60. Naftali-Shani N, Levin-Kotler L-P, Palevski D, Amit U, Kain D, Landa N, Hochhauser E, Leor J. Left Ventricular Dysfunction Switches Mesenchymal Stromal Cells Toward an Inflammatory Phenotype and Impairs Their Reparative Properties Via Toll-Like Receptor-4. *Circulation*. 2017;135:2271-2287. doi: 10.1161/circulationaha.116.023527
61. Ben-Mordechai T, Holbova R, Landa-Rouben N, Harel-Adar T, Feinberg MS, Abd Elrahman I, Blum G, Epstein FH, Silman Z, Cohen S, et al. Macrophage subpopulations are essential for infarct repair with and without stem cell therapy. *J Am Coll Cardiol*. 2013;62:1890-1901. doi: 10.1016/j.jacc.2013.07.057
62. Vagnozzi RJ, Maillet M, Sargent MA, Khalil H, Johansen AKZ, Schwanekamp JA, York AJ, Huang V, Nahrendorf M, Sadayappan S, et al. An acute immune response underlies the benefit of cardiac stem cell therapy. *Nature*. 2020;577:405-409. doi: 10.1038/s41586-019-1802-2
