# Supplementary material for: CRISPR-Cas9 editing of TLR4 to improve the outcome of cardiac cell therapy
Source: Sci Rep. 2023 Mar 18;13:4481. doi: 10.1038/s41598-023-31286-4 (PMC10024743; doi:10.1038/s41598-023-31286-4)
Supplement: Supplementary file 2 — Supplementary Information 2. [file 41598_2023_31286_MOESM2_ESM.pdf]

Supplementary Table 1.
Proteins identified with significant change

Protein names	Gene names	p value for change	Source	KEGG name
Macrophage colony-stimulating factor 1;Process	CSF1	0.0010	Growth media	Cytokine-cytokine receptor interaction;Hematopoietic cell lineage;Osteoclast differentiation;Rheumatoid arthritis
Decorin	DCN	0.0010	Growth media	TGF-beta signaling pathway
Collagen alpha-3(VI) chain	COL6A3	0.0010	Growth media	ECM-receptor interaction;Focal adhesion;Protein digestion and absorption
Myosin-9	MYH9	0.0010	Growth media	Tight junction;Viral myocarditis
Inhibin beta A chain	INHBA	0.0010	Growth media	Cytokine-cytokine receptor interaction;TGF-beta signaling pathway
Thymosin beta-10	TMSB10	0.0010	Growth media	
Eukaryotic initiation factor 4A-I;Eukaryotic initiat	EIF4A1;EIF4A2	0.0010	Growth media	RNA transport
Importin subunit beta-1	KPNB1	0.0010	Growth media	RNA transport
Ubiquitin-like modifier-activating enzyme 1	UBA1	0.0010	Growth media	Parkinson's disease;Ubiquitin mediated proteolysis
Transgelin-2	TAGLN2	0.0010	Growth media	
14-3-3 protein beta/alpha;14-3-3 protein beta/al	YWHAB	0.0010	Growth media	Cell cycle;Neurotrophin signaling pathway;Oocyte meiosis
Profilin-1	PFN1	0.0010	Growth media	Regulation of actin cytoskeleton;Shigellosis
Annexin A1	ANXA1	0.0010	Growth media	
14-3-3 protein theta	YWHAQ	0.0010	Growth media	Cell cycle;Neurotrophin signaling pathway;Oocyte meiosis
Prostaglandin F2 receptor negative regulator	PTGFRN	0.0010	Growth media	
Proteasome subunit beta type-6	PSMB6	0.0010	Growth media	Proteasome
Moesin	MSN	0.0010	Growth media	Leukocyte transendothelial migration;Measles;Regulation of actin cytoskeleton
Sclerostin	SOST	0.0010	Growth media	
	HLA-A	0.0010	Growth media	Allograft rejection;Antigen processing and presentation;Autoimmune thyroid disease;Cell adhesion molecules (CAMs);Endocytosis;Graft-versus-host disease;Natural killer cell mediated cytotoxicity;Phagosome;Type I diabetes mellitus;Viral myocarditis
Thioredoxin	TXN	0.0010	Growth media	
Ribonuclease inhibitor	RNH1	0.0010	Growth media	
Heat shock cognate 71 kDa protein	HSPA8	0.0010	Growth media	Antigen processing and presentation;Endocytosis;MAPK signaling pathway;Measles;Protein processing in endoplasmic reticulum;Spliceosome;Toxoplasmosis
Stathmin	STMN1	0.0010	Growth media	MAPK signaling pathway
Dihydropyrimidinase-related protein 2	DPYSL2	0.0010	Growth media	Axon guidance
Angiopoietin-related protein 4	ANGPTL4	0.0010	Growth media	PPAR signaling pathway
Elongation factor 1-delta	EEF1D	0.0010	Growth media	
Fascin	FSCN1	0.0010	Growth media	
Collagen alpha-1(XVIII) chain;Endostatin	COL18A1	0.0010	Growth media	Protein digestion and absorption
Glutathione S-transferase P	GSTP1	0.0010	Growth media	Drug metabolism - cytochrome P450;Glutathione metabolism;Metabolism of xenobiotics by cytochrome P450
Beta-hexosaminidase subunit beta;Beta-hexosan	HEXB	0.0010	Growth media	Amino sugar and nucleotide sugar metabolism;Glycosaminoglycan degradation;Glycosphingolipid biosynthesis - ganglio series;Glycosphingolipid biosynthesis - globo series;Lysosome;Other glycan degradation
Peroxiredoxin-6	PRDX6	0.0010	Growth media	Methane metabolism;Phenylalanine metabolism;Phenylpropanoid biosynthesis
Procollagen-lysine,2-oxoglutarate 5-dioxygenase	PLOD3	0.0010	Growth media	Lysine degradation;Other types of O-glycan biosynthesis
Elongation factor 1-gamma	EEF1G	0.0010	Growth media	
Myristoylated alanine-rich C-kinase substrate	MARCKS	0.0010	Growth media	Fc gamma R-mediated phagocytosis
Chloride intracellular channel protein 4	CLIC4	0.0010	Growth media	
Cysteine-rich protein 2	CRIP2	0.0010	Growth media	
	CALM2;CALM3;CAL	0.0010	Growth media	Alzheimer's disease;Calcium signaling pathway;Gastric acid secretion;Glioma;GnRH signaling pathway;Insulin signaling pathway;ko05152;Long-term potentiation;Melanogenesis;Neurotrophin signaling pathway;Olfactory transduction;Oocyte meiosis;Phosphatidylinositol signaling system;Phototransduction;Phototransduction - fly;Plant-pathogen interaction;Salivary secretion;Vascular smooth muscle contraction
Filamin-B	FLNB	0.0010	Growth media	Focal adhesion;MAPK signaling pathway
Transitional endoplasmic reticulum ATPase	VCP	0.0010	Growth media	Protein processing in endoplasmic reticulum
Cathepsin Z	CTSZ	0.0043	Growth media	Lysosome
60S acidic ribosomal protein P0-like;60S acidic ri	RPLP0;RPLP0P6	0.0044	Growth media	Ribosome
CD99 antigen	CD99	0.0045	Growth media	Cell adhesion molecules (CAMs);Leukocyte transendothelial migration
Latent-transforming growth factor beta-binding	LTBP1	0.0045	Growth media	
Phosphoglycerate kinase 1	PGK1	0.0046	Growth media	Carbon fixation in photosynthetic organisms;Glycolysis / Gluconeogenesis

Latent-transforming growth factor beta-binding p	LTBP3	0.0047	Growth media	
Protein S100;Protein S100-A6	S100A6	0.0048	Growth media	
Cysteine and glycine-rich protein 1	CSRP1	0.0048	Growth media	
5-nucleotidase	NT5E	0.0049	Growth media	Nicotinate and nicotinamide metabolism;Purine metabolism;Pyrimidine metabolism
Proteasome subunit beta type-5	PSMB5	0.0050	Growth media	Proteasome
Tropomyosin alpha-3 chain	TPM3	0.0051	Growth media	Cardiac muscle contraction;Dilated cardiomyopathy;Hypertrophic cardiomyopathy (HCM);Pathways in cancer;Thyroid cancer
Brain acid soluble protein 1	BASP1	0.0052	Growth media	
Fructose-bisphosphate aldolase C	ALDOC	0.0053	Growth media	Carbon fixation in photosynthetic organisms;Fructose and mannose metabolism;Glycolysis / Gluconeogenesis;Pentose phosphate pathway
Tissue factor pathway inhibitor 2	TFPI2	0.0054	Growth media	
F-actin-capping protein subunit alpha-1	CAPZA1	0.0055	Growth media	
Small ubiquitin-related modifier 4;Small ubiquitir	SUMO3;SUMO4;SU	0.0056	Growth media	RNA transport
Peroxidasin homolog	PXDN	0.0057	Growth media	
Adenylyl cyclase-associated protein 1;Adenylyl cy	CAP1	0.0058	Growth media	
Hepatocyte growth factor;Hepatocyte growth fac	HGF	0.0059	Growth media	Cytokine-cytokine receptor interaction;Focal adhesion;Malaria;Melanoma;Pathways in cancer;Renal cell carcinoma
Elongation factor 2	EEF2	0.0060	Growth media	
Procollagen C-endopeptidase enhancer 1	PCOLCE	0.0061	Growth media	
Procollagen-lysine,2-oxoglutarate 5-dioxygenase	PLOD2	0.0063	Growth media	Lysine degradation
Cathepsin L1;Cathepsin L1 heavy chain;Cathepsin	CTSL	0.0064	Growth media	Antigen processing and presentation;Lysosome;Phagosome;Rheumatoid arthritis
Integrin beta-like protein 1	ITGBL1	0.0065	Growth media	
Heat shock protein HSP 90-alpha	HSP90AA1	0.0067	Growth media	Antigen processing and presentation;NOD-like receptor signaling pathway;Pathways in cancer;Plant-pathogen interaction;Progesterone-mediated oocyte maturation;Prostate cancer;Protein processing in endoplasmic reticulum
Peptidyl-prolyl cis-trans isomerase A;Peptidyl-pro	PPIA	0.0068	Growth media	
Coronin-1C;Coronin	CORO1C	0.0070	Growth media	
Filamin-C	FLNC	0.0071	Growth media	Focal adhesion;MAPK signaling pathway
Collagen alpha-1(V) chain	COL5A1	0.0073	Growth media	Amoebiasis;ECM-receptor interaction;Focal adhesion;Protein digestion and absorption
Complement C1q tumor necrosis factor-related p	C1QTNF1	0.0075	Growth media	
Alpha-enolase	ENO1	0.0081	Growth media	Glycolysis / Gluconeogenesis;Methane metabolism;RNA degradation
Myosin light polypeptide 6;Myosin light chain 6B	PDE6H;MYL6;MYL6I	0.0082	Growth media	Vascular smooth muscle contraction
Translationally-controlled tumor protein	TPT1	0.0083	Growth media	
Adipocyte enhancer-binding protein 1	AEBP1	0.0085	Growth media	
Vascular endothelial growth factor A	VEGFA	0.0086	Growth media	Bladder cancer;Cytokine-cytokine receptor interaction;Focal adhesion;mTOR signaling pathway;Pancreatic cancer;Pathways in cancer;Renal cell carcinoma;Rheumatoid arthritis
Rab GDP dissociation inhibitor beta	GDI2	0.0097	Growth media	
Twisted gastrulation protein homolog 1	TWSG1	0.0098	Growth media	
EMILIN-1	EMILIN1	0.0100	Growth media	
Chloride intracellular channel protein 1	CLIC1	0.0101	Growth media	
Collagen alpha-1(XII) chain	COL12A1	0.0102	Growth media	Protein digestion and absorption
Nidogen-1	NID1	0.0103	Growth media	
Glutathione S-transferase omega-1	GSTO1	0.0104	Growth media	Drug metabolism - cytochrome P450;Glutathione metabolism;Metabolism of xenobiotics by cytochrome P450
Hemicentin-1	HMCN1	0.0105	Growth media	
Guanine nucleotide-binding protein G(I)/G(S)/G(T	GNB1	0.0107	Growth media	Chemokine signaling pathway;Phototransduction;Taste transduction
Eukaryotic translation initiation factor 5A-2;Euka	EIF5A2;EIF5A;EIF5A	0.0108	Growth media	
Collagen alpha-2(V) chain	COL5A2	0.0109	Growth media	Amoebiasis;ECM-receptor interaction;Focal adhesion;Protein digestion and absorption
	CTHRC1	0.0110	Growth media	
Phosphoglycerate mutase 1;Phosphoglycerate m	PGAM1;PGAM2;PG.	0.0112	Growth media	Glycolysis / Gluconeogenesis;Methane metabolism
WD repeat-containing protein 1	WDR1	0.0113	Growth media	
Transketolase	TKT	0.0115	Growth media	Biosynthesis of ansamycins;Carbon fixation in photosynthetic organisms;Pentose phosphate pathway
LIM and SH3 domain protein 1	LASP1	0.0116	Growth media	
Tubulin alpha-1A chain;Tubulin alpha-1C chain;Tu	TUBA1A;TUBA1C;TU	0.0117	Growth media	Gap junction;Pathogenic Escherichia coli infection;Phagosome
C-type lectin domain family 11 member A	CLEC11A	0.0119	Growth media	
Peroxiredoxin-1	PRDX1	0.0121	Growth media	Peroxisome
Filamin-A	FLNA	0.0122	Growth media	Focal adhesion;MAPK signaling pathway
Bone morphogenetic protein 1	BMP1	0.0143	Growth media	
14-3-3 protein gamma;14-3-3 protein gamma, N-	YWHAG	0.0144	Growth media	Cell cycle;Neurotrophin signaling pathway;Oocyte meiosis
Protein S100-A11;Protein S100-A11, N-terminally	S100A11	0.0146	Growth media	
Testican-1	SPOCK1	0.0147	Growth media	

High mobility group protein HMG-I/HMG-Y	HMGA1	0.0149	Growth media	
Nucleoside diphosphate kinase;Nucleoside dipho	NME2;NME1-NME2	0.0150	Growth media	Purine metabolism;Pyrimidine metabolism
Tripeptidyl-peptidase 1	TPP1	0.0152	Growth media	Lysosome
Alpha-actinin-4	ACTN4	0.0153	Growth media	Adherens junction;Amoebiasis;Arrhythmogenic right ventricular cardiomyopathy (ARVC);Focal adhesion;Leukocyte transendothelial migration;Regulation of actin cytoskeleton;Systemic lupus erythematosus;Tight junction
C-C motif chemokine 7	CCL7	0.0155	Growth media	Chemokine signaling pathway;Cytokine-cytokine receptor interaction
Polypeptide N-acetylgalactosaminyltransferase 2	GALNT2	0.0156	Growth media	Mucin type O-Glycan biosynthesis
Glutaminyl-peptide cyclotransferase	QPCT	0.0158	Growth media	
Dihydropyrimidinase-related protein 3	DPYSL3	0.0160	Growth media	
Annexin A2;Annexin;Putative annexin A2-like prc	ANXA2;ANXA2P2	0.0165	Growth media	
Thymosin beta-4;Hematopoietic system regulato	TMSB4X	0.0167	Growth media	Regulation of actin cytoskeleton
Malate dehydrogenase, mitochondrial;Malate de	MDH2	0.0168	Growth media	Carbon fixation in photosynthetic organisms;Citrate cycle (TCA cycle);Glyoxylate and dicarboxylate metabolism;Pyruvate metabolism
Cytokine receptor-like factor 1	CRLF1	0.0170	Growth media	
Fructose-bisphosphate aldolase A	ALDOA	0.0171	Growth media	Carbon fixation in photosynthetic organisms;Fructose and mannose metabolism;Glycolysis / Gluconeogenesis;Pentose phosphate pathway
Attractin	ATRN	0.0171	Growth media	
Metalloproteinase inhibitor 2	TIMP2	0.0172	Growth media	
Plastin-3	PLS3	0.0173	Growth media	
14-3-3 protein zeta/delta	YWHAZ	0.0173	Growth media	Cell cycle;Neurotrophin signaling pathway;Oocyte meiosis
Microtubule-associated protein;Microtubule-ass	MAP4	0.0174	Growth media	
Granulins;Acrogranin;Paragranulin;Granulin-1;Gr	GRN	0.0175	Growth media	
Integrin beta-1	ITGB1	0.0176	Growth media	Arrhythmogenic right ventricular cardiomyopathy (ARVC);Axon guidance;Bacterial invasion of epithelial cells;Cell adhesion molecules (CAMs);Dilated cardiomyopathy;ECM-receptor interaction;Focal adhesion;Hypertrophic cardiomyopathy (HCM);Leishmaniasis;Leukocyte transendothelial migration;Pathogenic Escherichia coli infection;Pathways in cancer;Phagosome;Regulation of actin cytoskeleton;Shigellosis;Small cell lung cancer;Toxoplasmosis
Clathrin heavy chain 1;Clathrin heavy chain 2	CLTC;CLTCL1	0.0176	Growth media	Bacterial invasion of epithelial cells;Endocrine and other factor-regulated calcium reabsorption;Endocytosis;Huntington's disease;Lysosome
Putative heat shock protein HSP 90-beta 2	HSP90AB2P	0.0177	Growth media	
Urokinase plasminogen activator surface recepto	PLAUR	0.0178	Growth media	Complement and coagulation cascades
L-lactate dehydrogenase B chain;L-lactate dehyd	LDHB	0.0179	Growth media	Cysteine and methionine metabolism;Glycolysis / Gluconeogenesis;Propanoate metabolism;Pyruvate metabolism
Prelamin-A/C;Lamin-A/C	LMNA	0.0180	Growth media	Arrhythmogenic right ventricular cardiomyopathy (ARVC);Dilated cardiomyopathy;Hypertrophic cardiomyopathy (HCM)
C-C motif chemokine 2	CCL2	0.0182	Growth media	Chagas disease (American trypanosomiasis);Chemokine signaling pathway;Cytokine-cytokine receptor interaction;Malaria;NOD-like receptor signaling pathway;Rheumatoid arthritis
Protein DJ-1	PARK7	0.0183	Growth media	Parkinson's disease
Cell migration-inducing and hyaluronan-binding p	CEMIP	0.0185	Growth media	
Nucleobindin-1	NUCB1	0.0186	Growth media	
60S acidic ribosomal protein P2	RPLP2	0.0186	Growth media	Ribosome
Cathepsin B;Cathepsin B light chain;Cathepsin B I	CTSB	0.0188	Growth media	Antigen processing and presentation;Lysosome
Galectin-1	LGALS1	0.0188	Growth media	
NKG2D ligand 2	ULBP2	0.0189	Growth media	Natural killer cell mediated cytotoxicity
Soluble scavenger receptor cysteine-rich domain	SSC5D	0.0190	Growth media	
CD59 glycoprotein	CD59	0.0191	Growth media	Complement and coagulation cascades;Hematopoietic cell lineage
Malate dehydrogenase;Malate dehydrogenase, c	MDH1	0.0192	Growth media	Carbon fixation in photosynthetic organisms;Citrate cycle (TCA cycle);Glyoxylate and dicarboxylate metabolism;Proximal tubule bicarbonate reclamation;Pyruvate metabolism
Collagen alpha-2(VI) chain	COL6A2	0.0194	Growth media	ECM-receptor interaction;Focal adhesion;Protein digestion and absorption
Procollagen-lysine,2-oxoglutarate 5-dioxygenase	PLOD1	0.0208	Growth media	Lysine degradation
Plectin	PLEC	0.0209	Growth media	
Glucose-6-phosphate isomerase	GPI	0.0211	Growth media	Amino sugar and nucleotide sugar metabolism;Glycolysis / Gluconeogenesis;Pentose phosphate pathway;Starch and sucrose metabolism
Ras GTPase-activating-like protein IQGAP1	IQGAP1	0.0212	Growth media	Regulation of actin cytoskeleton
Basigin	BSG	0.0213	Growth media	
ERO1-like protein alpha	ERO1L	0.0215	Growth media	Protein processing in endoplasmic reticulum;Vibrio cholerae infection
Glyceraldehyde-3-phosphate dehydrogenase	GAPDH	0.0216	Growth media	Alzheimer's disease;Glycolysis / Gluconeogenesis
Submaxillary gland androgen-regulated protein 3	SMR3B	0.0218	Growth media	

Zyxin	ZYX	0.0219	Growth media	Focal adhesion
Cadherin-11	CDH11	0.0221	Growth media	
Triosephosphate isomerase	TPI1	0.0222	Growth media	Carbon fixation in photosynthetic organisms;Fructose and mannose metabolism;Glycolysis / Gluconeogenesis;Inositol phosphate metabolism
Ribonuclease 4	RNASE4	0.0224	Growth media	
Integrin beta;Integrin beta-5	ITGB5	0.0225	Growth media	Arrhythmogenic right ventricular cardiomyopathy (ARVC);Dilated cardiomyopathy;ECM-receptor interaction;Focal adhesion;Hypertrophic cardiomyopathy (HCM);Phagosome;Regulation of actin cytoskeleton
L-lactate dehydrogenase A chain	LDHA	0.0227	Growth media	Cysteine and methionine metabolism;Glycolysis / Gluconeogenesis;Propanoate metabolism;Pyruvate metabolism
Alpha-actinin-1	ACTN1	0.0229	Growth media	Adherens junction;Amoebiasis;Arrhythmogenic right ventricular cardiomyopathy (ARVC);Focal adhesion;Leukocyte transendothelial migration;Regulation of actin cytoskeleton;Systemic lupus erythematosus;Tight junction
Stanniocalcin-1	STC1	0.0230	Growth media	
Complement factor H	CFH	0.0231	Growth media	Complement and coagulation cascades;Staphylococcus aureus infection
Immunoglobulin superfamily containing leucine-r	ISLR	0.0232	Growth media	
Pigment epithelium-derived factor	SERPINF1	0.0233	Growth media	
Tubulin beta chain;Tubulin beta-2B chain;Tubulin	TUBB;TUBB2B;TUBE	0.0234	Growth media	Gap junction;Pathogenic Escherichia coli infection;Phagosome
Atrial natriuretic peptide receptor 3	NPR3	0.0234	Growth media	
Prosaposin;Saposin-A;Saposin-B-Val;Saposin-B;Sa	PSAP	0.0235	Growth media	Lysosome
Integrin alpha-2	ITGA2	0.0235	Growth media	Arrhythmogenic right ventricular cardiomyopathy (ARVC);Dilated cardiomyopathy;ECM-receptor interaction;Focal adhesion;Hematopoietic cell lineage;Hypertrophic cardiomyopathy (HCM);Pathways in cancer;Phagosome;Regulation of actin cytoskeleton;Small cell lung cancer
Laminin subunit beta-1	LAMB1	0.0237	Growth media	Amoebiasis;ECM-receptor interaction;Focal adhesion;Pathways in cancer;Small cell lung cancer;Toxoplasmosis
Endoglin	ENG	0.0238	Growth media	
Heterogeneous nuclear ribonucleoprotein A1;He	HNRNPA1	0.0239	Growth media	Spliceosome
Laminin subunit gamma-2	LAMC2	0.0240	Growth media	Amoebiasis;ECM-receptor interaction;Focal adhesion;Pathways in cancer;Small cell lung cancer;Toxoplasmosis
Pyruvate kinase PKM;Pyruvate kinase	PKM	0.0241	Growth media	Carbon fixation in photosynthetic organisms;Glycolysis / Gluconeogenesis;Purine metabolism;Pyruvate metabolism;Type II diabetes mellitus
Transmembrane protein 132A	TMEM132A	0.0241	Growth media	
Nidogen-2	NID2	0.0242	Growth media	
Lactadherin;Lactadherin short form;Medin	MFGE8	0.0242	Growth media	
Disintegrin and metalloproteinase domain-conta	ADAM10	0.0243	Growth media	Alzheimer's disease;Epithelial cell signaling in Helicobacter pylori infection
Exostosin-2	EXT2	0.0244	Growth media	Glycosaminoglycan biosynthesis - heparan sulfate
Microfibrillar-associated protein 2	MFAP2	0.0245	Growth media	
60S acidic ribosomal protein P1	RPLP1	0.0245	Growth media	Ribosome
Growth-regulated alpha protein;GRO-alpha(4-73	CXCL1	0.0245	Growth media	Chemokine signaling pathway;Cytokine-cytokine receptor interaction
Dickkopf-related protein 3	DKK3	0.0246	Growth media	
Renin receptor	ATP6AP2	0.0246	Growth media	
Collagen alpha-2(I) chain	COL1A2	0.0246	Growth media	Amoebiasis;ECM-receptor interaction;Focal adhesion;Protein digestion and absorption
Fibulin-2	FBLN2	0.0247	Growth media	
Plasminogen activator inhibitor 1	SERPINE1	0.0248	Growth media	Chagas disease (American trypanosomiasis);Complement and coagulation cascades;p53 signaling pathway
78 kDa glucose-regulated protein	HSPA5	0.0248	Growth media	Prion diseases;Protein export;Protein processing in endoplasmic reticulum
Protein disulfide-isomerase	P4HB	0.0249	Growth media	Protein processing in endoplasmic reticulum
Cofilin-1	CFL1	0.0249	Growth media	Axon guidance;Fc gamma R-mediated phagocytosis;Regulation of actin cytoskeleton
Cytoskeleton-associated protein 4	CKAP4	0.0250	Growth media	Protein processing in endoplasmic reticulum
Semaphorin-7A	SEMA7A	0.0251	Growth media	Axon guidance
Complement C1s subcomponent;Complement C1	C1S	0.0251	Growth media	Complement and coagulation cascades;Staphylococcus aureus infection;Systemic lupus erythematosus
C-X-C motif chemokine;Interleukin-8;MDNCF-a;Ir	CXCL8	0.0253	Growth media	Amoebiasis;Bladder cancer;Chagas disease (American trypanosomiasis);Chemokine signaling pathway;Cytokine-cytokine receptor interaction;Epithelial cell signaling in Helicobacter pylori infection;Hepatitis C;Malaria;NOD-like receptor signaling pathway;Pathways in cancer;Rheumatoid arthritis;RIG-I-like receptor signaling pathway;Shigellosis;Toll-like receptor signaling pathway
Calsyntenin-1;Soluble Alc-alpha;CTF1-alpha	CLSTN1	0.0253	Growth media	

Collagen alpha-2(IV) chain;Canstatin	COL4A2	0.0254	Growth media	Amoebiasis;ECM-receptor interaction;Focal adhesion;Pathways in cancer;Protein digestion and absorption;Small cell lung cancer
Heterogeneous nuclear ribonucleoproteins A2/B	HNRNPA2B1	0.0254	Growth media	
EGF-like repeat and discoidin I-like domain-conta	EDIL3	0.0254	Growth media	
Interleukin-6	IL6	0.0255	Growth media	African trypanosomiasis;Amoebiasis;Chagas disease (American trypanosomiasis);Cytokine-cytokine receptor interaction;Cytosolic DNA-sensing pathway;Graft-versus-host disease;Hematopoietic cell lineage;Hypertrophic cardiomyopathy (HCM);Intestinal immune network for IgA production;Jak-STAT signaling pathway;ko05152;Malaria;Measles;NOD-like receptor signaling pathway;Pathways in cancer;Prion diseases;Rheumatoid arthritis;Toll-like receptor signaling pathway
Integrin alpha-V;Integrin alpha-V heavy chain;Int	ITGAV	0.0256	Growth media	Arrhythmogenic right ventricular cardiomyopathy (ARVC);Cell adhesion molecules (CAMs);Dilated cardiomyopathy;ECM-receptor interaction;Focal adhesion;Hypertrophic cardiomyopathy (HCM);Pathways in cancer;Phagosome;Regulation of actin cytoskeleton;Small cell lung cancer
CD9 antigen	CD9	0.0257	Growth media	Hematopoietic cell lineage
Spectrin alpha chain, non-erythrocytic 1	SPTAN1	0.0257	Growth media	
CMP-N-acetylneuraminate-beta-galactosamide-a	ST3GAL1	0.0258	Growth media	Glycosaminoglycan biosynthesis - keratan sulfate;Glycosphingolipid biosynthesis - ganglio series;Glycosphingolipid biosynthesis - globo series;Mucin type O-Glycan biosynthesis
Prolyl 3-hydroxylase 1	LEPRE1	0.0259	Growth media	
ADAMTS-like protein 1	ADAMTSL1	0.0259	Growth media	
Versican core protein	VCAN	0.0261	Growth media	Cell adhesion molecules (CAMs)
Collagen alpha-1(I) chain	COL1A1	0.0261	Growth media	Amoebiasis;ECM-receptor interaction;Focal adhesion;Protein digestion and absorption
Collagen alpha-1(XVI) chain	COL16A1	0.0262	Growth media	
Thioredoxin reductase 1, cytoplasmic	GML;TXNRD1	0.0262	Growth media	Pyrimidine metabolism;Selenocompound metabolism
Out at first protein homolog	OAF	0.0264	Growth media	
Interleukin-11	IL11	0.0271	Growth media	Cytokine-cytokine receptor interaction;Hematopoietic cell lineage;Jak-STAT signaling pathway;Rheumatoid arthritis
Aminopeptidase N	ANPEP	0.0273	Growth media	Glutathione metabolism;Hematopoietic cell lineage;Renin-angiotensin system
Cadherin-2	CDH2	0.0274	Growth media	Arrhythmogenic right ventricular cardiomyopathy (ARVC);Cell adhesion molecules (CAMs)
Latent-transforming growth factor beta-binding p	LTBP2	0.0275	Growth media	
Follistatin-related protein 1	FSTL1	0.0276	Growth media	
Tropomyosin alpha-4 chain	TPM4	0.0278	Growth media	Cardiac muscle contraction;Dilated cardiomyopathy;Hypertrophic cardiomyopathy (HCM)
Heat shock 70 kDa protein 6;Putative heat shock	HSPA6;HSPA7	0.0279	Growth media	Antigen processing and presentation;Endocytosis;MAPK signaling pathway;Measles;Protein processing in endoplasmic reticulum;Spliceosome;Toxoplasmosis
Pappalysin-1	PAPPA	0.0280	Growth media	
ADM;Adrenomedullin;Proadrenomedullin N-20 t	ADM	0.0281	Growth media	Vascular smooth muscle contraction
14-3-3 protein sigma	SFN	0.0282	Growth media	Aldosterone-regulated sodium reabsorption;Cell cycle;p53 signaling pathway
Secreted frizzled-related protein 1	SFRP1	0.0283	Growth media	Wnt signaling pathway
Granulocyte colony-stimulating factor	CSF3	0.0283	Growth media	Cytokine-cytokine receptor interaction;Hematopoietic cell lineage;Jak-STAT signaling pathway;Malaria
Agrin;Agrin N-terminal 110 kDa subunit;Agrin C-t	AGRN	0.0283	Growth media	ECM-receptor interaction
Metalloproteinase inhibitor 1	TIMP1	0.0284	Growth media	
Metallothionein-2;Metallothionein-1X;Metalloth	MT2A;MT1X;MT1G	0.0284	Growth media	Mineral absorption
Glypican-1;Secreted glypican-1	GPC1	0.0284	Growth media	
Thrombospondin-2	THBS2	0.0285	Growth media	ECM-receptor interaction;Focal adhesion;Malaria;Phagosome;TGF-beta signaling pathway
Insulin-like growth factor-binding protein 4	IGFBP4	0.0286	Growth media	
Sushi repeat-containing protein SRPX2	SRPX2	0.0286	Growth media	
Transforming growth factor-beta-induced protei	TGFBI	0.0287	Growth media	
Peptidyl-prolyl cis-trans isomerase FKBP10;Pepti	FKBP10	0.0287	Growth media	
Putative elongation factor 1-alpha-like 3;Elongati	EEF1A1P5;EEF1A1	0.0288	Growth media	RNA transport
Nucleobindin-2;Nesfatin-1	DCAF5;NUCB2;Nucl	0.0289	Growth media	
Caldesmon	CALD1	0.0290	Growth media	Vascular smooth muscle contraction
Annexin A5;Annexin	ANXA5	0.0290	Growth media	
Glucosidase 2 subunit beta	PRKCSH	0.0291	Growth media	Protein processing in endoplasmic reticulum
Amyloid beta A4 protein;N-APP;Soluble APP-alph	APP	0.0291	Growth media	Alzheimer's disease
Sushi repeat-containing protein SRPX	SRPX	0.0292	Growth media	
Hedgehog-interacting protein	HHIP	0.0293	Growth media	Basal cell carcinoma;Hedgehog signaling pathway;Pathways in cancer
Gelsolin	GSN	0.0294	Growth media	Fc gamma R-mediated phagocytosis;Regulation of actin cytoskeleton

Heat shock protein HSP 90-beta	HSP90AB1	0.0294	Growth media	Antigen processing and presentation;NOD-like receptor signaling pathway;Pathways in cancer;Plant-pathogen interaction;Progesterone-mediated oocyte maturation;Prostate cancer;Protein processing in endoplasmic reticulum
Interstitial collagenase;22 kDa interstitial collage	MMP1	0.0295	Growth media	Bladder cancer;Pathways in cancer;PPAR signaling pathway;Rheumatoid arthritis
Laminin subunit gamma-1	LAMC1	0.0295	Growth media	Amoebiasis;ECM-receptor interaction;Focal adhesion;Pathways in cancer;Prion diseases;Small cell lung cancer;Toxoplasmosis
Biglycan	BGN	0.0296	Growth media	
Laminin subunit alpha-5	LAMA5	0.0297	Growth media	Amoebiasis;ECM-receptor interaction;Focal adhesion;Pathways in cancer;Small cell lung cancer;Toxoplasmosis
Stanniocalcin-2	STC2	0.0301	Growth media	
Insulin-like growth factor-binding protein 7	IGFBP7	0.0302	Growth media	
Prothymosin alpha;Prothymosin alpha, N-termin	PTMA	0.0303	Growth media	
Protein disulfide-isomerase A6	PDIA6	0.0304	Growth media	Protein processing in endoplasmic reticulum
Peptidyl-prolyl cis-trans isomerase B	PPIB	0.0306	Growth media	
10 kDa heat shock protein, mitochondrial	HSPE1;HSPE1-MOB	0.0307	Growth media	
Calumenin	CALU	0.0308	Growth media	
Peptidyl-prolyl cis-trans isomerase FKBP1A;Pepti	FKBP1A;FKBP12-Exi	0.0315	Growth media	
Ubiquitin carboxyl-terminal hydrolase isozyme L1	UCHL1	0.0316	Growth media	Parkinson's disease
Neuropilin-1	NRP1	0.0318	Growth media	Axon guidance
Complement C4-A;Complement C4 beta chain;Co	C4A;C4B	0.0319	Growth media	Complement and coagulation cascades;Staphylococcus aureus infection;Systemic lupus erythematosus
Connective tissue growth factor	CTGF	0.0320	Growth media	
EGF-containing fibulin-like extracellular matrix pr	EFEMP2	0.0321	Growth media	
SPARC	SPARC	0.0323	Growth media	
Ubiquitin-60S ribosomal protein L40;Ubiquitin;60	UBB;RPS27A;UBC;U	0.0323	Growth media	Parkinson's disease;PPAR signaling pathway;Ribosome
Protein disulfide-isomerase A3	PDIA3	0.0324	Growth media	Antigen processing and presentation;Protein processing in endoplasmic reticulum
Meteorin-like protein	METRNL	0.0324	Growth media	
Uncharacterized protein KIAA0930	KIAA0930	0.0325	Growth media	
Protein CYR61	CYR61	0.0325	Growth media	
Talin-1	TLN1	0.0332	Growth media	Focal adhesion
Fibulin-1	FBLN1	0.0333	Growth media	
45 kDa calcium-binding protein	SDF4	0.0334	Growth media	
4F2 cell-surface antigen heavy chain	SLC3A2	0.0358	Growth media	Protein digestion and absorption
Cystatin-C	CST3	0.0380	Growth media	Salivary secretion
Basement membrane-specific heparan sulfate pr	HSPG2	0.0391	Growth media	ECM-receptor interaction
Glia-derived nexin	SERPINE2	0.0402	Growth media	
Serpin H1	SERPINH1	0.0410	Growth media	
Transforming growth factor beta-1;Latency-assoc	TGFB1	0.0411	Growth media	Amoebiasis;Cell cycle;Chagas disease (American trypanosomiasis);Chronic myeloid leukemia;Colorectal cancer;Cytokine-cytokine receptor interaction;Dilated cardiomyopathy;Endocytosis;Hypertrophic cardiomyopathy (HCM);Intestinal immune network for IgA production;ko05152;Leishmaniasis;Malaria;MAPK signaling pathway;Osteoclast differentiation;Pancreatic cancer;Pathways in cancer;Renal cell carcinoma;Rheumatoid arthritis;TGF-beta signaling pathway;Toxoplasmosis
Transgelin	TAGLN	0.0425	Growth media	
Thioredoxin domain-containing protein 5	TXNDC5	0.0427	Growth media	Protein processing in endoplasmic reticulum
Laminin subunit alpha-4	LAMA4	0.0429	Growth media	African trypanosomiasis;Amoebiasis;ECM-receptor interaction;Focal adhesion;Pathways in cancer;Small cell lung cancer;Toxoplasmosis
Thrombospondin-4	THBS4	0.0430	Growth media	ECM-receptor interaction;Focal adhesion;Malaria;Phagosome;TGF-beta signaling pathway
Complement C1r subcomponent;Complement C1	C1R	0.0432	Growth media	Complement and coagulation cascades;Phagosome;Staphylococcus aureus infection;Systemic lupus erythematosus
Endoplasmin	HSP90B1	0.0436	Growth media	NOD-like receptor signaling pathway;Pathways in cancer;Plant-pathogen interaction;Prostate cancer;Protein processing in endoplasmic reticulum
Aldo-keto reductase family 1 member C1;Aldo-ke	AKR1C2;AKR1C1;AK	0.0436	Growth media	Arachidonic acid metabolism;Metabolism of xenobiotics by cytochrome P450;Steroid hormone biosynthesis
Cathepsin D;Cathepsin D light chain;Cathepsin D	CTSD	0.0437	Growth media	ko05152;Lysosome
Protein disulfide-isomerase A4	PDIA4	0.0438	Growth media	Protein processing in endoplasmic reticulum;Vibrio cholerae infection
Cadherin-13	CDH13	0.0440	Growth media	
Integral membrane protein 2B;BRI2, membrane	ITM2B	0.0442	Growth media	
Macrophage migration inhibitory factor	MIF	0.0443	Growth media	Phenylalanine metabolism;Tyrosine metabolism
Guanine nucleotide-binding protein G(I)/G(S)/G(	GNG12	0.0445	Growth media	Chemokine signaling pathway;MAPK signaling pathway;Regulation of actin cytoskeleton

Dystroglycan;Alpha-dystroglycan;Beta-dystroglyc	DAG1	0.0447	Growth media	Arrhythmogenic right ventricular cardiomyopathy (ARVC);Dilated cardiomyopathy;ECM-receptor interaction;Hypertrophic cardiomyopathy (HCM);Viral myocarditis
Collagen alpha-1(VII) chain	COL7A1	0.0450	Growth media	
72 kDa type IV collagenase;PEX	MMP2	0.0459	Growth media	Bladder cancer;GnRH signaling pathway;Leukocyte transendothelial migration;Pathways in cancer
Lysosomal Pro-X carboxypeptidase	PRCP	0.0469	Growth media	Protein digestion and absorption
Proteasome subunit alpha type;Proteasome subu	PSMA6	0.0472	Growth media	Proteasome
Tubulin beta-4B chain;Tubulin beta-4A chain	TUBB4B;TUBB4A	0.0499	Growth media	Gap junction;Pathogenic Escherichia coli infection;Phagosome

Supplementary Table 1.
Proteins identified with significant change

Protein names	Gene names	p value for change	Source	KEGG name
Protein S100-A6	S100A6	0.0007	Evs	
Myristoylated alanine-rich C-kinase substrate	MARCKS	0.0064	Evs	Fc gamma R-mediated phagocytosis
Putative elongation factor 1-alpha-like 3;Elongati	EEF1A1P5;EEF1A1	0.0076	Evs	RNA transport
Versican core protein	VCAN	0.0127	Evs	Cell adhesion molecules (CAMs)
Laminin subunit gamma-1	LAMC1	0.0146	Evs	Amoebiasis;ECM-receptor interaction;Focal adhesion;Pathways in cancer;Prion diseases;Small cell lung cancer;Toxoplasmosis
Brain acid soluble protein 1	BASP1	0.0147	Evs	
Thrombospondin-2	THBS2	0.0270	Evs	ECM-receptor interaction;Focal adhesion;Malaria;Phagosome;TGF-beta signaling pathway
Actin, cytoplasmic 2;Actin, cytoplasmic 1;Actin, g	ACTG1;ACTB	0.0303	Evs	Adherens junction;Arrhythmogenic right ventricular cardiomyopathy (ARVC);Bacterial invasion of epithelial cells;Cardiac muscle contraction;Dilated cardiomyopathy;Focal adhesion;Hypertrophic cardiomyopathy (HCM);Leukocyte transendothelial migration;Pathogenic Escherichia coli infection;Phagosome;Phototransduction - fly;Regulation of actin cytoskeleton;Shigellosis;Tight junction;Vascular smooth muscle contraction;Vibrio cholerae infection;Viral myocarditis
MARCKS-related protein	MARCKSL1	0.0304	Evs	Fc gamma R-mediated phagocytosis;Leishmaniasis
14-3-3 protein zeta/delta	YWHAZ	0.0400	Evs	Cell cycle;Neurotrophin signaling pathway;Oocyte meiosis
Desmoglein-1	DSG1	0.0401	Evs	Staphylococcus aureus infection
EMILIN-1	EMILIN1	0.0415	Evs	
Heat shock cognate 71 kDa protein	HSPA8	0.0446	Evs	Antigen processing and presentation;Endocytosis;ko05152;MAPK signaling pathway;Measles;Protein processing in endoplasmic reticulum;RNA degradation;Spliceosome;Toxoplasmosis
