# Supplementary material for: CRISPR-Cas9 editing of TLR4 to improve the outcome of cardiac cell therapy
Source: Sci Rep. 2023 Mar 18;13:4481. doi: 10.1038/s41598-023-31286-4 (PMC10024743; doi:10.1038/s41598-023-31286-4)

Supplementary Table 2.

Echocardiography variables before and after MI.

	Edited (n=9)	Unedited (n=10)	Saline (n=14)	*p* 2-way RM ANOVA
**Area; Systole (mm²)**				
**Baseline**	5.68±2.5	5.98±4.56	5.28±1.29	*p* Interaction= 0.6
**Day 1 after MI**	6.79±2.04	8.26±4.26	6.38±1.8	*p* Time< 0.0001
**Day 28 after MI**	8.84±4.07	12.13±10.05	10.83±4.84	*p* Treatment=0.28
***p* day 1 vs. 28 (Holm-Šídák)**	0.108	0.086	0.008	
**Area; Diastole (mm²)**				
**Baseline**	10.22±2.11	11.48±4.61	10.11±1.1	*p* Interaction= 0.92
**Day 1 after MI**	9.2±1.9	11.67±5.47	9.15±1.7	*p* Time< 0.0001
**Day 28 after MI**	12.75±3.65	15.68±10	13.92±5.11	*p* Treatment=0.09
***p* day 1 vs. 28 (Holm-Šídák)**	0.011	0.039	0.01	
**Volume; Systole (µL)**				
**Baseline**	22.47±12.41	17.3±9.62	20.97±4.54	*p* Interaction= 0.01
**Day 1 after MI**	30.59±11.45	29.37±6.98	29.24±8.75	*p* Time= < 0.0001
**Day 28 after MI**	39.14±11.6	46.69±29.88	63.9±28.35	*p* Treatment=0.05
***p* day 1 vs. 28 (Holm-Šídák)**	0.039	0.05	0.0005	
**Volume; Diastole (µL)**				
**Baseline**	48.99±17.79	42.1±14.37	48.66±6.03	*p* Interaction= 0.2
**Day 1 after MI**	43.09±13.8	43.79±9.56	43.09±8.95	*p* Time< 0.0001
**Day 28 after MI**	59.4±14.44	67.22±29.37	78.49±29.23	*p* Treatment=0.18
***p* day 1 vs. 28 (Holm-Šídák)**	0.006	0.005	0.001	

	Edited (n=9)	Unedited (n=10)	Saline (n=14)	*p* 2-way RM ANOVA
**Ejection Fraction (%)**				
**Baseline**	54.82±15.56	59.62±14.78	56.94±6.62	*p* Interaction= 0.06
**Day 1 after MI**	29.17±12.71	32.92±10.49	32.1±12.98	*p* Time< 0.0001
**Day 28 after MI**	33.77±11.16	32.92±17.26	19.85±10	*p* Treatment=0.005
***p* day 1 vs. 28 (Holm-Šídák)**	0.217	0.999	0.007	
**Diameter; Systole (mm)**				
**Baseline**	2.14±0.77	2.09±0.69	2.41±0.36	*p* Interaction= 0.59
**Day 1 after MI**	2.82±0.37	2.7±0.61	2.69±0.31	*p* Time< 0.0001
**Day 28 after MI**	3.22±0.7	3.39±1.19	3.61±0.71	*p* Treatment=0.26
***p* day 1 vs. 28 (Holm-Šídák)**	0.151	0.047	0.0002	
**Diameter; Diastole (mm)**				
**Baseline**	3.3±0.61	3.39±0.41	3.42±0.27	*p* Interaction= 0.41
**Day 1 after MI**	3.39±0.43	3.54±0.84	3.18±0.28	*p* Time< 0.0001
**Day 28 after MI**	4.02±0.67	4.24±1.03	4.02±0.69	*p* Treatment=0.25
***p* day 1 vs. 28 (Holm-Šídák)**	0.038	0.005	0.0008	
**Fractional Shortening (%)**				
**Baseline**	35.97±13.29	38.93±12.97	29.81±6.71	*p* Interaction= 0.2
**Day 1 after MI**	16.61±9.98	15.96±8.03	15.42±5.74	*p* Time< 0.0001
**Day 28 after MI**	19.83±11.19	18.88±11.93	10.5±4.39	*p* Treatment< 0.0001
***p* day 1 vs. 28 (Holm-Šídák)**	0.154	0.34	0.005	

	Edited (n=9)	Unedited (n=10)	Saline (n=14)	*p* 2-way RM ANOVA
**LV Mass (*corrected*) (mg)**				
**Baseline**	66.22±28.8	74.64±19.94	65.15±9.66	*p* Interaction= 0.27
**Day 1 after MI**	79.43±15.99	60.64±13.66	72.49±19.88	*p* Time< 0.0001
**Day 28 after MI**	109.66±70.82	99.73±48.08	93.97±24.77	*p* Treatment=0.43
***p* day 1 vs. 28 (Holm-Šídák)**	0.153	0.004	0.025	
**LVAW; Systole (mm)**				
**Baseline**	1.11±0.24	1.21±0.23	1.11±0.14	*p* Interaction= 0.06
**Day 1 after MI**	1.12±0.21	0.91±0.25	1.03±0.22	*p* Time= 0.01
**Day 28 after MI**	1.06±0.54	0.96±0.22	0.8±0.37	*p* Treatment=0.07
***p* day 1 vs. 28 (Holm-Šídák)**	0.952	0.373	0.083	
**LVAW; Diastole (mm)**				
**Baseline**	0.78±0.15	0.82±0.12	0.71±0.11	*p* Interaction= 0.13
**Day 1 after MI**	0.96±0.25	0.74±0.13	0.86±0.21	*p* Time= 0.14
**Day 28 after MI**	0.83±0.3	0.82±0.18	0.73±0.3	*p* Treatment=0.07
***p* day 1 vs. 28 (Holm-Šídák)**	0.381	0.213	0.288	
**LVPW; Systole (mm)**				
**Baseline**	1.1±0.11	1.22±0.27	1.03±0.13	*p* Interaction= 0.1
**Day 1 after MI**	0.97±0.31	0.9±0.27	1.01±0.25	*p* Time= 0.01
**Day 28 after MI**	1.16±0.5	1.01±0.13	1±0.16	*p* Treatment=0.56
***p* day 1 vs. 28 (Holm-Šídák)**	0.156	0.224	0.903	

	Edited (n=9)	Unedited (n=10)	Saline (n=14)	*p* 2-way RM ANOVA
**LVPW; Diastole (mm)**				
**Baseline**	0.75±0.16	0.82±0.28	0.77±0.12	*p* Interaction= 0.04
**Day 1 after MI**	0.77±0.33	0.68±0.16	0.86±0.18	*p* Time= 0.03
**Day 28 after MI**	0.9±0.32	0.75±0.09	0.86±0.14	*p* Treatment= 0.05
***p* day 1 vs. 28 (Holm-Šídák)**	0.413	0.085	0.997	

Supplementary Table 3.

Donor patients' basic clinical characteristics

**Serial number**	C3	C5	C6	C7
**Sex (male=1 female=2)**	1	1	1	2
**Age**	63	71	49	71
**BMI**	21.61	22.09	29.35	25.39
**Hypertension (no=o yes=1)**	0	1	1	1
**Dyslipidemia (no=o yes=1)**	0	1	1	1
**Diabetes (no=o yes=1)**	0	0	0	0
**Smoking (no=o yes=1)**	1	0	0	0
**Diabetes**	No	No	No	No

Supplementary Table 4.

Electrical and other parameters examined for the electroporation reaction. In **BOLD** final parameters chosen. The bottom part of the chart illustrates the meaning of some of the electrical parameters tested.

Number of pulses	Voltage (Volts, x100)	Pulse time (ms)	Interval time (ms)	Secondary pulse voltage (Volts)	Secondary pulse time (ms)	Medium	Number of cells (x10^5)	Cuvette size (mm)	Reaction volume (µL)	gRNA:Cas9 ratio for preparing RNP	Number of gRNA tested
**1** / 2 / 3 / 5 / 8	0.3 / 0.6 / **1.25** / 1.5 / 2 / 3 / 4 / 5 / 6	0.1 / 3 / **5** / 60 / 75 / 100 / 125 / 300	**0** / 250 / 750 / 5000	**0** / 15 / 20 / 25 / 30	**0** / 100 / 150 / 200	Growth medium / **DMEM** / PBS / Opti-MEM	**1** / 2 / 3 / 5 / 7.5 / 10	4 / 2 / **1**	500 / 400 / 50 / **80**	1:1 / 2:1 / **3:1**	**4**

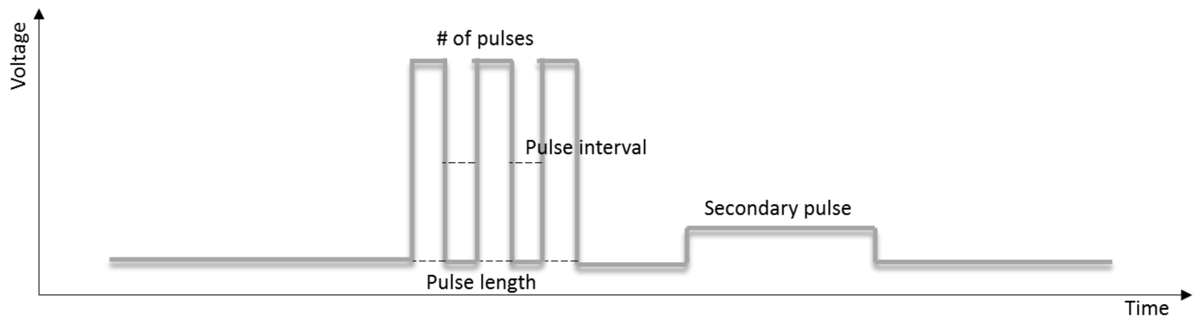

Supplement: Supplementary file 3 — Supplementary Information 3. [file 41598_2023_31286_MOESM3_ESM.pdf]
